# Cytotoxic Anthranilic Acid Derivatives from Deep Sea Sediment-Derived Fungus *Penicillium paneum* SD-44

**DOI:** 10.3390/md11083068

**Published:** 2013-08-21

**Authors:** Chun-Shun Li, Xiao-Ming Li, Shu-Shan Gao, Yan-Hua Lu, Bin-Gui Wang

**Affiliations:** 1 Key Laboratory of Experimental Marine Biology, Institute of Oceanology, Chinese Academy of Sciences, Nanhai Road 7, Qingdao 266071, China; E-Mails: lichunshun@ms.qdio.ac.cn (C.-S.L.); lixmqd@yahoo.com.cn (X.-M.L.); xisea01@126.com (S.-S.G.); 2 State Key Laboratory of Bioreactor Engineering, East China University of Science & Technology, Shanghai 200237, China; E-Mail: luyanhua@ecust.edu.cn

**Keywords:** marine fungus, sediment, anthranilic acid, *Penicillium paneum*, cytotoxicity

## Abstract

Five new anthranilic acid derivatives, penipacids A–E (**1**–**5**), together with one known analogue (**6**), which was previously synthesized, were characterized from the ethyl acetate extract of the marine sediment-derived fungus *Penicillium paneum* SD-44. Their structures were elucidated mainly by extensive NMR spectroscopic and mass spectrometric analysis. The cytotoxicity and antimicrobial activity of the isolated compounds were evaluated. Compounds **1**, and **5** exhibited inhibitory activity against human colon cancer RKO cell line, while compound **6** displayed cytotoxic activity against Hela cell line.

## 1. Introduction

**Figure 1:**
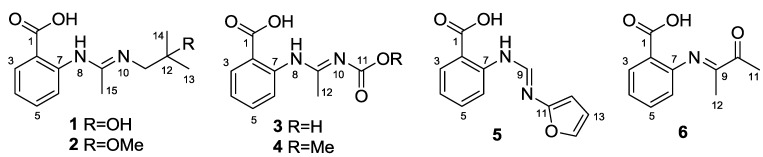
Structures of the isolated compounds **1**–**6**.

Marine fungi have recently attracted great attention owing to their structurally unique and biologically active metabolites [1,2,3,4]. Our previous investigation of filamentous fungi from marine habitats [5,6,7,8,9,10] enabled us to obtain a fungus *Penicillium paneum* SD-44 from the sediment sample collected from the South China Sea. Chemical investigation of this fungus by static culture in solid rice medium led to the isolation of one novel triazole and two new quinazolinone alkaloids, penipanoids A–C [11]. During our ongoing exploration of new bioactive metabolites of this fungal strain by changing fermentation conditions, we had a chance to access a large-scale bioreactor, and, as a result, six anthranilic acid derivatives including five new ones (**1**–**5**) and one previously synthesized analogue (**6**) [12] (Figure 1) were isolated from the culture broth of the dynamic fermentation in a 500-L fermentator. All the isolated new compounds possess an amidine moiety, which is rare among naturally occurring compounds [13]. Details of the isolation, structure elucidation, and biological activities are reported herein.

## 2. Results and Discussion

### Structure Elucidation of the New Compounds

**Figure 2:**
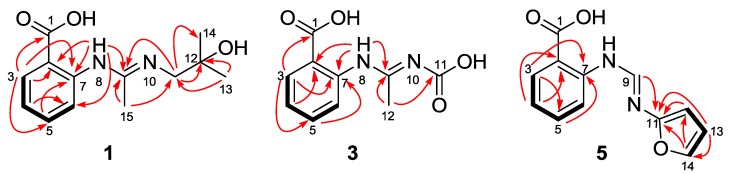
Key HMBC (arrows) and ^1^H–^1^H COSY (bold lines) correlations of compounds **1**, **3**, and **5**.

Penipacid A (**1**) was isolated as yellowish solid. Its molecular formula was demonstrated as C_13_H_18_N_2_O_3_ by HR-ESI-MS, with six degrees of unsaturation. Detailed analyses of the 1D NMR data (Table 1 and Table 2) indicated the presence of one carbonyl, one *ortho*-disubstituted benzene ring, two additional quaternary carbons (one sp^2^ and one sp^3^), one methylene carbon and three methyls. Among them, the de-shielded sp^3^ quaternary carbon resonating at *δ*_C_ 70.8 was deduced to be oxygenated, while the two sp^2^ carbons at *δ*_C_ 148.3 and 148.4 and the methylene at *δ*_C_ 49.7 were ascribed to be connected with nitrogen atoms. The characters of the 1D NMR data and the UV spectrum (λ_max_ 286 and 340 nm) as well as the observed ^1^H–^1^H COSY and HMBC correlations (Figure 2) suggested that **1** might be an anthranilic acid derivative [14]. In the HMBC spectrum, the correlation from the double doublet aromatic proton H-3 to C-1 (*δ*_C_ 172.9) allowed the placement of the carboxyl group at C-2 (Figure 2). The observed HMBC cross-peaks from the exchangeable proton (H-8, *δ*_H_ 10.63), ascribed to nitrogenated atom, to the aromatic carbons C-2 and C-6 implied the presence of the 2-aminobenzoic acid moiety. Meanwhile, the HMBC correlations (Figure 2) from the symmetrical methyls (H_3_-13 and H_3_-14) to the oxygenated carbon C-12 (*δ*_C_ 70.8) and the nitrogen-bearing methylene C-11 (*δ*_C_ 49.7) and from singlet methyl H_3_-15 to C-9 (*δ*_C_ 148.4/3) and C-11 as well as from H-8 to C-9 suggested the presence of *N'*-(2-hydroxy-2-methylpropyl)acetimidamide motif in the structure of **1**. The observed NOE correlation of H_2_-11 with H_3_-15 ascribed the imine double bond C_9_=N_10_ to be *E*-configured. The structure of **1** was thus established to be (*E*)-2-(*N'*-(2-hydroxy-2-methylpropyl)acetimidamido)benzoic acid, named as penipacid A. 

Penipacid B (**2**), yellowish powder, was revealed by HR-ESI-MS data to have the molecular formula C_14_H_20_N_2_O_3_, with a CH_2_ unit more than that of **1**. Comparison of the NMR data of **2** with those of **1** indicated that the structures of these two compounds are very similar, except that one methoxy group was present in **2**. The resonance of the oxygenated quaternary carbon C-12 shifted downfield from *δ*_C_ 70.8 in **1** to *δ*_C_ 76.8 in **2**, while the adjacent methyl carbon C-13/C-14 moved upfield from *δ*_C_ 29.4 in **1** to *δ*_C_ 25.6 in the ^13^C NMR spectrum of **2** (Table 2). Correspondingly, the methyl signal H-13/H-14 shifted from *δ*_H_ 1.35 in **1** to upfield at *δ*_H_ 1.23 in the ^1^H NMR spectrum of **2** (Table 1). The observation implied that the hydroxy group at C-12 in **1** was replaced by a methoxy moiety in **2**, which was verified by the obvious HMBC correlation from the protons of the methoxy (*δ*_H_ 3.27) to C-12. The NOE correlation of H_2_-11 with H_3_-15 also suggested the *E*-configured of C_9_=N_10_. Thus, the structure of **2** was established as (*E*)-2-(*N'*-(2-methoxy-2-methylpropyl)acetimidamido)benzoic acid.

**Table 1:** ^1^H NMR data for compounds **1**–**6** (500 MHz, *δ* in ppm, *J* in Hz).

No.	1 *^a^*	2 *^b^*	3 *^c^*	4 *^b^*	5 *^b^*	6 *^b^*
3	7.97 dd (7.9, 0.8)	7.89 dd (8.0, 1.3)	7.89 d (7.9)	7.98 d (7.7)	7.92 d (7.8)	7.99 dd (7.9, 1.2)
4	6.76 br t (7.9)	6.70 br t (8.0)	6.94 t (7.4)	6.93 t (7.5)	6.77 t (7.5)	6.94 td (8.0, 0.9)
5	7.45 td ( 8.2, 1.0)	7.38 td (8.5, 1.4)	7.55 t (7.4)	7.48 t (7.5)	7.42 t (7.5)	7.46 td (8.0, 1.5)
6	7.50 br d (8.2)	7.58 br d (8.5)	7.81 d (8.4)	7.80 d (8.4)	7.66 br d (8.5)	7.76 br d (8.4)
8	10.63 s	-	11.36 s	-	-	
9					7.82 s	
11	2.53 s	2.53 s				2.47 s
12			2.04 s	2.14 s	6.63 d (3.1)	2.01 s
13	1.35 s	1.23 s			6.50 dd (2.8, 1.7)	
14	1.35 s	1.23 s			7.55 br s	
15	2.00 s	1.97 s				
OMe		3.27 s				

*^a^* Recorded in CDCl_3_; *^b^* Recorded in methanol-*d*_4_; *^c^* Recorded in DMSO-*d*_6_.

**Table 2:** ^13^C NMR data for compounds **1**–**6** (125 MHz, δ in ppm).

No.	1 *^a^*	2 *^b^*	3 *^c^*	4 *^b^*	5 *^b^*	6 *^b^*
1	172.9 s	172.3 s *^d^*	170.4 s	172.7 s	172.2 s	173.3 s *^d^*
2	108.4 s	111.1 s	112.4 s	112.1 s	112.2 s	117.6 s
3	131.8 d	132.5 d	131.6 d	132.6 d	132.5 d	132.7 d
4	117.5 d	117.9 d	120.2 d	121.0 d	118.7 d	121.3 d
5	135.7 d	135.2 d	135.0 d	135.0 d	135.1 d	134.2 d
6	113.1 d	114.1 d	114.3 d	115.0 d	114.4 d	114.5 d
7	148.3 s	149.7 s	146.4 s	147.5 s	148.7 s	146.8 s
9	148.4 s	147.9 s	136.5 s	135.4 s	131.5 d	143.4 s
10						199.3 s
11	49.7 t	48.7 t	166.2 s	167.4 s	152.3 s	8.5 q
12	70.8 s	76.8 s	11.4 q	11.4 q	110.8 d	24.2 q
13	29.4 q	25.6 q			112.7 d	
14	29.4 q	25.6 q			144.5 d	
15	17.9 q	17.3 q				
OMe		49.0 q		52.7 q		

*^a^* Recorded in CDCl_3_; *^b^* Recorded in methanol-*d*_4_; *^c^* Recorded in DMSO-*d*_6_; *^d^* Data deduced from HMBC.

Penipacid C (**3**) was shown to have the molecular formula of C_10_H_10_N_2_O_4_ (seven degrees of unsaturation) by means of HR-ESI-MS. Detailed analysis of the ^1^H-, ^13^C- as well as DEPT NMR data of **3** suggested that the molecule might possess the same 2-acetimidamido benzoic acid moiety as those of compounds **1** and **2**. The remaining structural unit (COOH) with the quaternary carbon atom resonated at *δ*_C_ 166.2 (C-11) was ascribed to the presence of one carbonyl group. The obvious HMBC correlations from the only methyl group (*δ*_H_ 2.04, H_3_-12) to C-9 (*δ*_C_ 136.5) and C-11 established the position of the remaining carbonyl group (Figure 2). The chemical atmosphere of the methyl (C-12) might be influenced by the carboxyl group (C-11), which was correspondingly affected by the configuration of the double bond (C_9_=N_10_). When the C_9_=N_10_ takes the *E* configuration, the methyl group (C-12) is at the shielding area of the carboxyl group, which implied the lower chemical shift of C-12 in this mode than in that of the *Z* configured C_9_=N_10_ molecule. The preliminary prediction of the chemical shift of C-12 by the software ChemBioDraw Ultra (V12.0) verified this deduction that C-12 was in a higher fielded atmosphere (*δ*_C_ 18.9) in the *E*-configured molecule, and in a lower one (*δ*_C_ 24.9) for the *Z*-configured model. The experimental value (*δ*_C_ 11.4) of the chemical shift of C-12 is closer to that of the predicted one of *E*-configured molecule than that of the *Z*-configured model. The configuration of the double bond C_9_=N_10_ was thus tentatively assigned to be *E*, which was biogenetically identical to those of compounds **1** and **2**. A double bond isomer (C_9_=N_10_) of **3** was previously described in a complex with manganese [15].

Penipacid E (**4**) was isolated as yellowish solid. Its molecular formula was determined to be C_11_H_12_N_2_O_4_ by HR-ESI-MS, possessing one more CH_2_ moiety than that of **3**. Comprehensive comparison of the 1D NMR data of **4** with those of **3** suggested that the two molecules were very similar, except that an *O*-methyl group (*δ*_H_ 3.84 and *δ*_C_ 52.7) was observed in the NMR spectra of **4**, implying one of the COOH group in **3** was replaced by a COOMe group in **4**. The HMBC correlation from the methoxy group to C-11 (*δ*_C_ 167.4) allowed the placement of the COOMe group at N-10. The geometry of the double bond (C_9_=N_10_) in **4** was also assigned by the chemical shift prediction using ChemBioDraw Ultra (version 12.0) software to be *E*-configured. The similarity of the chemical shifts of compounds **3** and **4** also implied that both of them should take the same configuration. 

Penipacid G (**5**) was obtained as a yellowish solid. The molecular formula was deduced from the HR-ESI-MS data to be C_12_H_10_N_2_O_3_, with nine degrees of unsaturation. The similarity of the 1D NMR data of **5** with compounds **1**–**4** suggested that **5** should also be an anthranilic acid derivative. Besides the 2-aminobenzoic acid moiety, the remaining part (C_5_H_4_NO) of the structure was established by 2D NMR spectra. The COSY correlations from H-12 to H-13 and from H-13 to H-14 as well as the HMBC correlations from H-12 and H-13 to the oxygenated aromatic carbons C-11 (*δ*_C_ 152.3) and C-14 (*δ*_C_ 144.5) and from H-14 to C-11 and C-12 (Figure 2) implied the presence of the nitrogen substituted furan moiety. Moreover, the HMBC cross peak from H-9 to C-11 established the planar structure of compound **5** to be 2-(*N'*-(furan-2-yl)formimidamido)benzoic acid. The NOE correlation from H-9 to the furan proton H-12 implied the *E*-configuration of C_9_=N_10_.

To clarify whether the new compounds **2** and **4** are artifacts, which might be respectively derived from **1** and **3** during the purification procedures, compounds **1** and **3** were mixed with silica gel in CHCl_3_–MeOH (1:1) for 24 hours and then checked by HPLC. Additionally, these compounds were respectively dissolved in MeOH and were stirred at room temperature for 48 hours and then also analyzed by HPLC. The results indicated that none of them showed obvious change compared with the standard samples. Based on the above experiments, compounds **2** and **4** are regarded as natural occurring products, rather than the artifacts.

Compounds **1**–**6** were evaluated for the cytotoxicity against Hela and RKO cell lines and the antimicrobial activity on two bacteria (*Staphylococcus aureus* and *Escherichia coli*) and three plant-pathogenic fungi (*Alternaria brassicae*, *Fusarium graminearum*, and *Rhizoctonia cerealis*). In the cytotoxic assays, penipacids A (**1**) and E (**5**) exhibited inhibitory activity against RKO cell line with an IC_50_ value of 8.4 and 9.7 µM, respectively, while compound **6** displayed cytotoxic activity against Hela cell line with the IC_50_ value of 6.6 µM, which are all stronger than the positive control fluorouracil (with IC_50_ values of 25.0 and 14.5 µM, respectively). In the antimicrobial screening, no obvious activity could be observed for the tested compounds.

## 3. Experimental Section

### 3.1. General

UV Spectroscopic data were obtained on a Lengguang Gold S54. NMR Spectra were recorded at 500 and 125 MHz for ^1^H and ^13^C, respectively, on a Bruker Advance 500. Mass spectra were measured on a VG Autospec 3000 mass spectrometer. Column chromatography (CC) was performed with silica gel (200–300 mesh, Qingdao Marine Chemical Factory, Qingdao, China), Lobar LiChroprep RP-18 (40–63 µm; Merck), and Sephadex LH-20 (18–110 µm, Merck, Darmstadt, Germany). HPLC was performed using an Elite semi-preparative colume (10 × 300 mm, Elite, Dalian, China) on Dionex HPLC system.

### 3.2. Fungal Material

The procedures of isolation and identification of the fungal strain used in this experiment were described in an earlier report [11].

### 3.3. Extraction and Isolation

The fresh mycelium was inoculated into 500 mL flask preloaded with 200 mL liquid medium (consisting of mannitol 20 g, maltose 20 g, monosodium glutamate 10 g, glucose 10 g, yeast extract 3 g, corn steep liquor 1 g, KH_2_PO_4_ 0.5 g, and MgSO_4_·7H_2_O 0.3 g, in 1 L filtered sea water) followed by a two-day culture incubation at 28 °C and 150 rpm/min. The whole liquid (15 × 200 mL) collected from the flask was inoculated into a 50-L seed fermentator containing 27 L sterilized medium for a one-day fermentation at 28 °C and 150 rpm/min. The cultured liquid was then transferred into a 500 L fermentator preloaded with 270 L sterilized medium and cultured for another 5 days at the same conditions.

The fermented mycelia and broth were separated by centrifugation, and the mycelia were exhaustively extracted with acetone to afford the crude extract (70.0 g). The extract was fractionated by Si gel vacuum liquid chromatography (VLC) using petroleum ether (PE)–EtOAc (from 1:0 to 1:1) and CHCl_3_–MeOH (from 20:1 to 0:1) gradient elution to afford 10 fractions (Fr.1–Fr.10). Fr.2 (6.1 g) eluted with PE–EtOAc (5:1) was further purified by column chromatography (CC) on reversed-phase silica gel C_18_ eluted with a MeOH–H_2_O gradient (20% to 100%) to obtain eight parts (P.1–P.8). P.4 (170.0 mg) was further purified by semi-preparative HPLC (Elite ODS-BP column, 10 μm; 10.0 × 300 mm; 3 mL/min; 70% MeOH/H_2_O with 0.1% acetic acid in mobile phases) to obtain compounds **4** (5.7 mg, *t*_R_ 10.2 min), **1** (16.7 mg, *t*_R_ 17.5 min), and **2** (9.3 mg, *t*_R_ 27.6 min;). P.5 (110.0 mg) was then subjected to Sephadex LH-20 (MeOH), which was followed by semi-preparative HPLC (73% MeOH/H_2_O) to get **5** (4.1 mg, *t*_R_ 19.6 min). P.6 (180.0 mg) was fractionated by Sephadex LH-20 (MeOH) and semi-preparative HPLC to yield compound **6 **(8.7 mg, *t*_R_ 15.5 min; 65% MeOH/H_2_O). Fr.3 (8.43 g) eluted with PE–EtOAc (2:1) was further separated by CC on reversed-phase silica gel C_18_ eluted with a MeOH–H_2_O gradient (20% to 100%) to afford eight parts (P.1–P.8). P.7 (125.0 mg) was separated by semi-preparative HPLC (75% MeOH/H_2_O) to get **3** (21.2 mg, *t*_R_ 9.8 min).

*Penipacid A* (**1**): yellowish solid; UV (MeOH) λ_max_ (log ε) 215 (4.50), 286 (4.40), 340 (3.85) nm; ^1^H and ^13^C NMR data, see Table 1 and Table 2; ESIMS *m/z* 251 [M + H]^+^; HRESIMS *m/z* 251.1393 [M + H]^+^ (calcd for C_13_H_19_N_2_O_3_, 251.1390).

*Penipacid B* (**2**): yellowish powder; UV (MeOH) λ_max_ (log ε) 215 (4.06), 286 (4.07), 336 (3.48) nm; ^1^H and ^13^C NMR data, see Table 1 and Table 2; ESIMS *m/z* 287 [M + Na]^+^; HRESIMS *m/z* 287.1365 [M + Na]^+^ (calcd for C_14_H_20_N_2_O_3_Na, 287.1371).

*Penipacid C* (**3**): yellowish solid; UV (MeOH) λ_max_ (log ε) 218 (4.70) nm, 334 (4.66); ESIMS *m/z* 223 [M + H]^+^; HRESIMS *m/z* 223.0713 (calcd for C_10_H_11_N_2_O_4_, 223.0714). 

*Penipacid* *D* (**4**): yellowish solid; UV (MeOH) λ_max_ (log ε) 217 (4.31) nm, 334 (4.42); ^1^H and ^13^C NMR data, see Table 1 and Table 2; ESIMS *m/z* 237 [M + H]^+^; HRESIMS *m/z* 237.0869 [M + H]^+^ (calcd for C_11_H_13_N_2_O_4_, 237.0870).

*PenipacidE* (**5**): yellowish solid; UV (MeOH) λ_max_ (log ε) 215 (4.65), 350 (4.75) nm; ^1^H and ^13^C NMR data, see Table 1 and Table 2, respectively; ESIMS *m/z* 229 [M − H]^−^; HRESIMS *m/z* 229.0629 [M − H]^−^ (calcd for C_12_H_9_N_2_O_3_, 229.0619).

### 3.4. Cytotoxic Assay

The cytotoxic activity against Hela (human epithelial carcinoma) and RKO (human colon cancer) cell lines was determined according to previously reported methods [16]. Briefly, cells were seeded onto 96-well plates at a density of 4 × 10^3^ cells/well for 24 h, and treated with gradient concentrations of the tested compounds for 48 h. MTT (100 µL, 0.5 mg/mL) was added to each well and the cells were incubated for further 4 h in the dark at 37 °C. Then, the dye crystals were dissolved in 150 µL dimethyl sulphoxide (DMSO) after careful removal of the medium. Absorbance was measured at 570 nm using a microplate reader (BioTek, USA). The viability of the treated groups was assessed as a percentage of non-treated control groups, which was assumed to be 100%. The cytotoxicity of the compounds was expressed as an IC_50_, defined as the concentration causing a 50% reduction of cell growth compared with untreated cells.

### 3.5. Antimicrobial Assays

The antimicrobial activities against two bacteria (*S. aureus* and *E. coli*) and three plant-pathogenic fungi (*A. brassicae*, *F. graminearum*, and *R. cerealis*) were carried out using the disk diffusion method [17]. Chloramphenicol and amphotericin B were used as antibacterial and antifungal positive controls, respectively.

## 4. Conclusions

Five new anthranilic acid derivatives, penipacids A–E (**1**–**5**), along with one known analogue (**6**), were identified from *P. paneum* SD-44 fermented in a 500 L bioreactor. Compounds **1**, **5**, and **6** exhibited cytotoxic activities. The biosynthetic potential of filamentous fungi is proven to be under exploited [18,19]. The strategy of changing culture conditions of fungi for inducing the production of new metabolites has been successfully applied in recent years [20,21,22,23]. Detailed HPLC-DAD analysis of the crude extract and VLC fractions of fungus SD-44 cultured in rice medium revealed that none of the penipacid compounds could be detected. However, the penipanoids, which were isolated from the static fermentation [11] were also isolated from the dynamic cultured products in the present investigation. Since there is no more powerful evidence to prove whether the penipacid compounds could be metabolized by SD-44 in the static fermentation, the discovery of penipacids could not be definitely ascribed to the change of fermentation condition of the fungus SD-44. 

## Supplementary Files

Supplementary File 1 Supplementary Materials (PDF, 1906 KB)
