# Supplementary material for: Cytotoxic Anthranilic Acid Derivatives from Deep Sea Sediment-Derived Fungus Penicillium paneum SD-44
Source: Mar Drugs. 2013 Aug 21;11(8):3068–76. doi: 10.3390/md11083068 (PMC3766882; doi:10.3390/md11083068)

Supplementary Materials

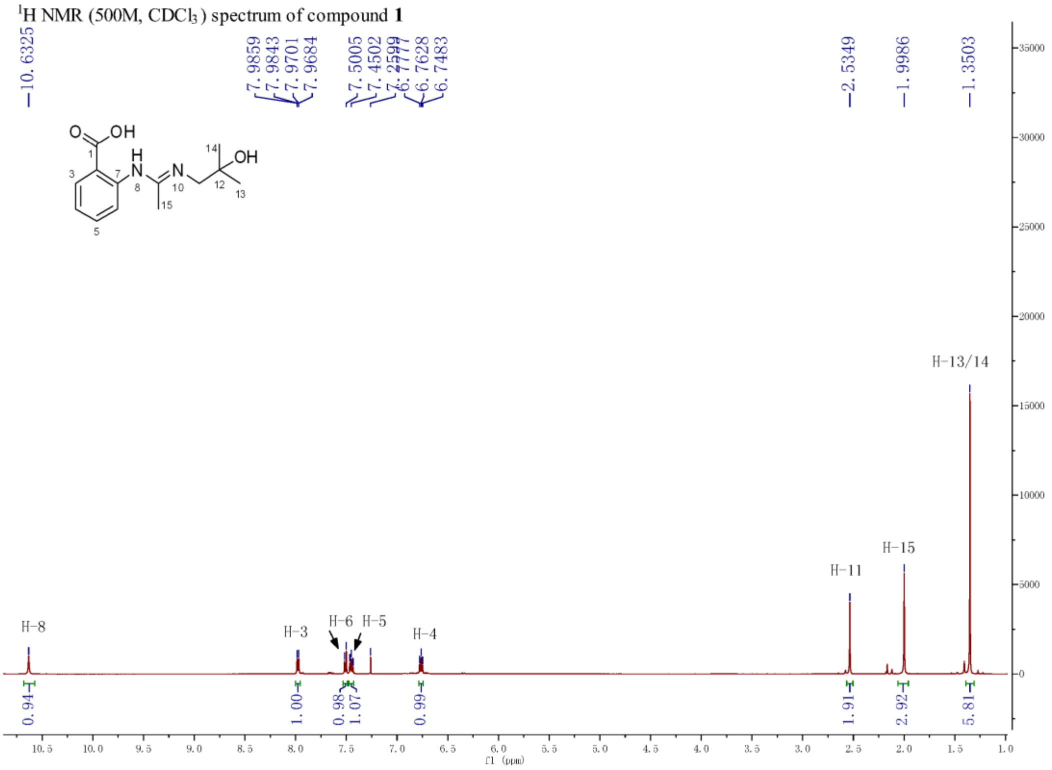

Figure S1. ^{1}H NMR (500 MHz, $CDCl_3$) spectrum of compound **1**.

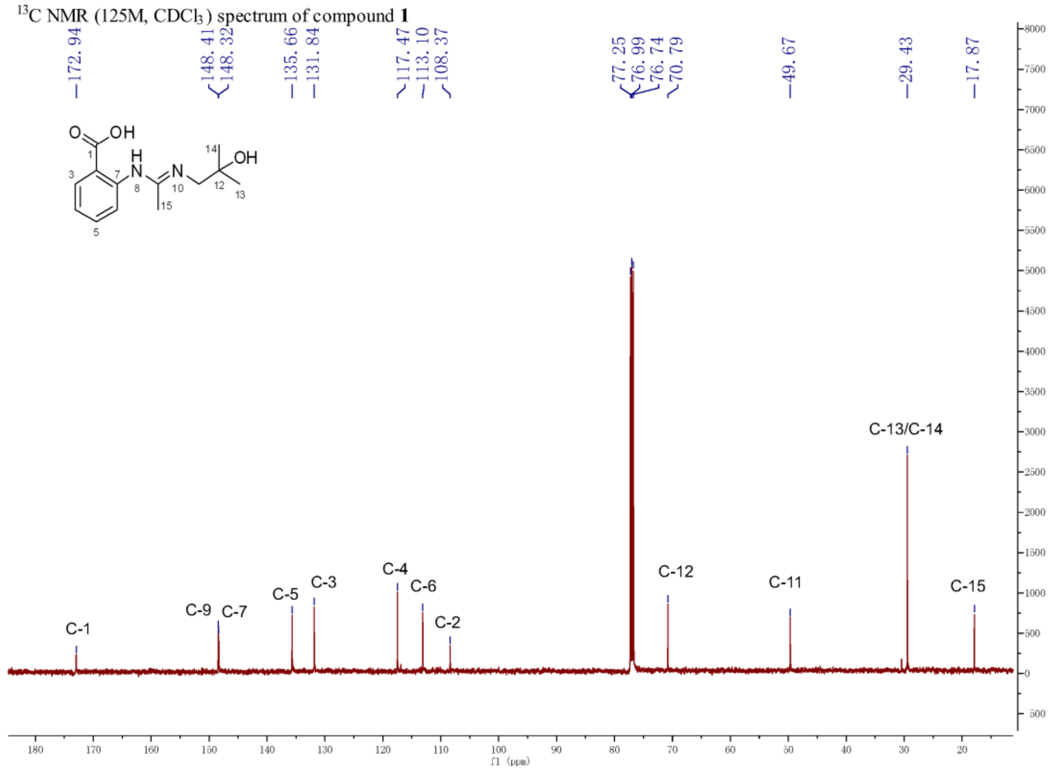

Figure S2. ^{13}C NMR (125 MHz, $CDCl_3$) spectrum of compound **1**.

Figure S3. $^{1}H–^{1}H$ COSY spectrum of compound **1**.

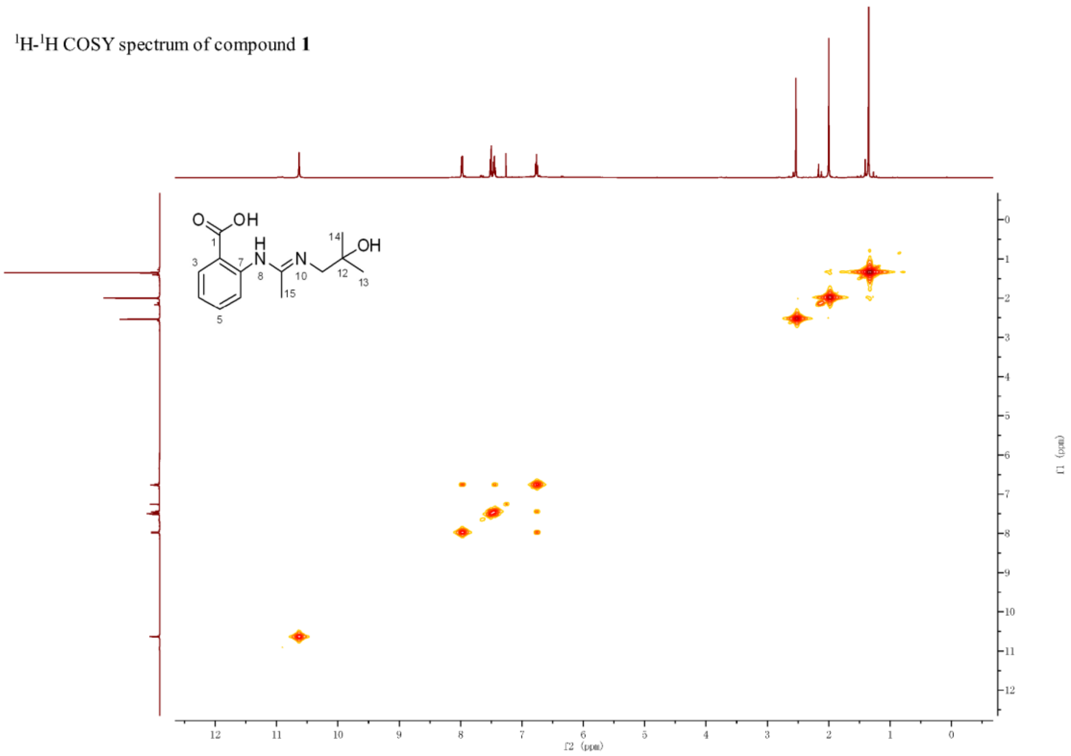

Figure S4. HSQC spectrum of compound **1**.

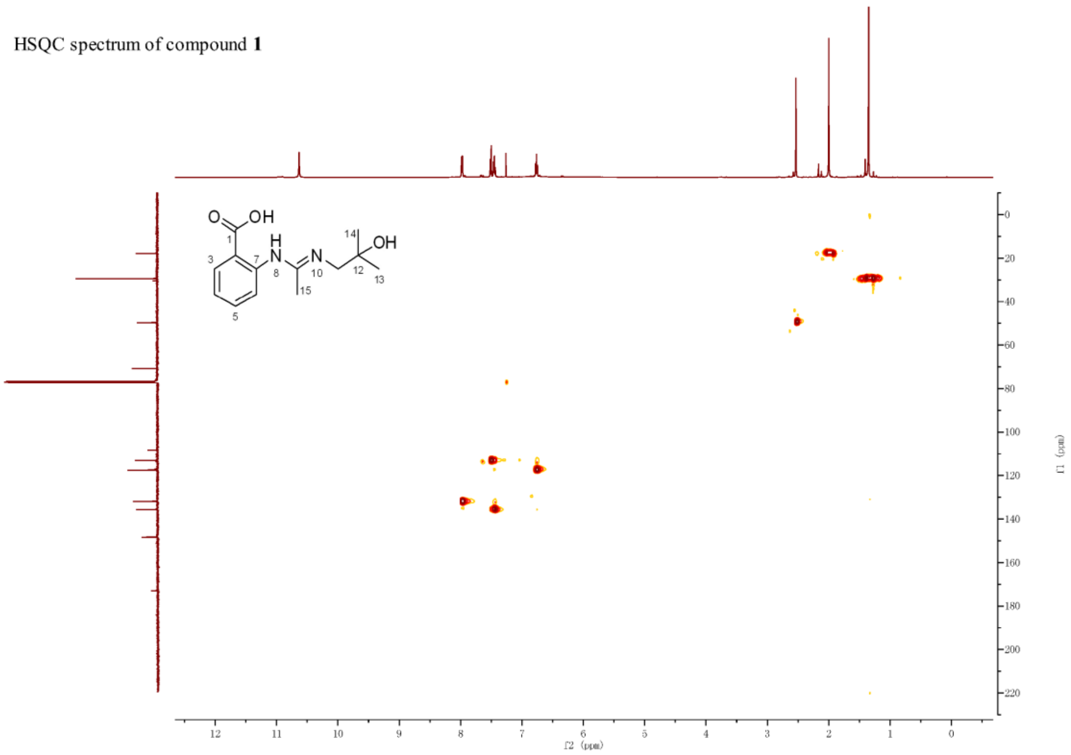

Figure S5. HMBC spectrum of compound **1**.

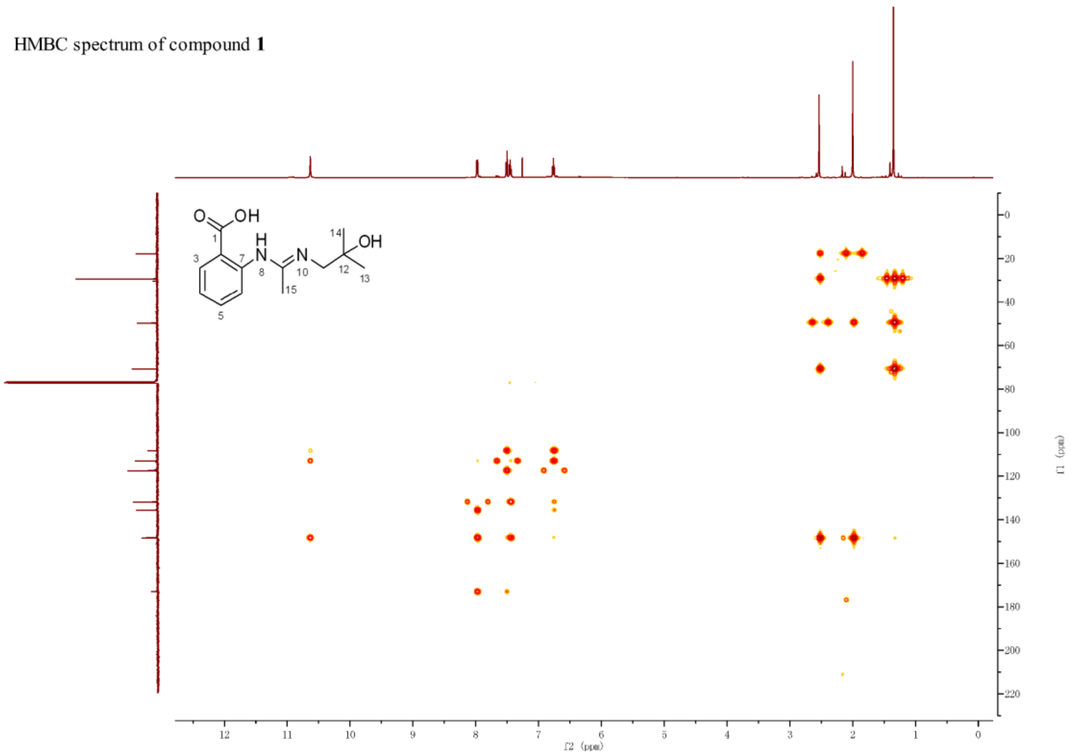

Figure S6. NOESY spectrum of compound **1**.

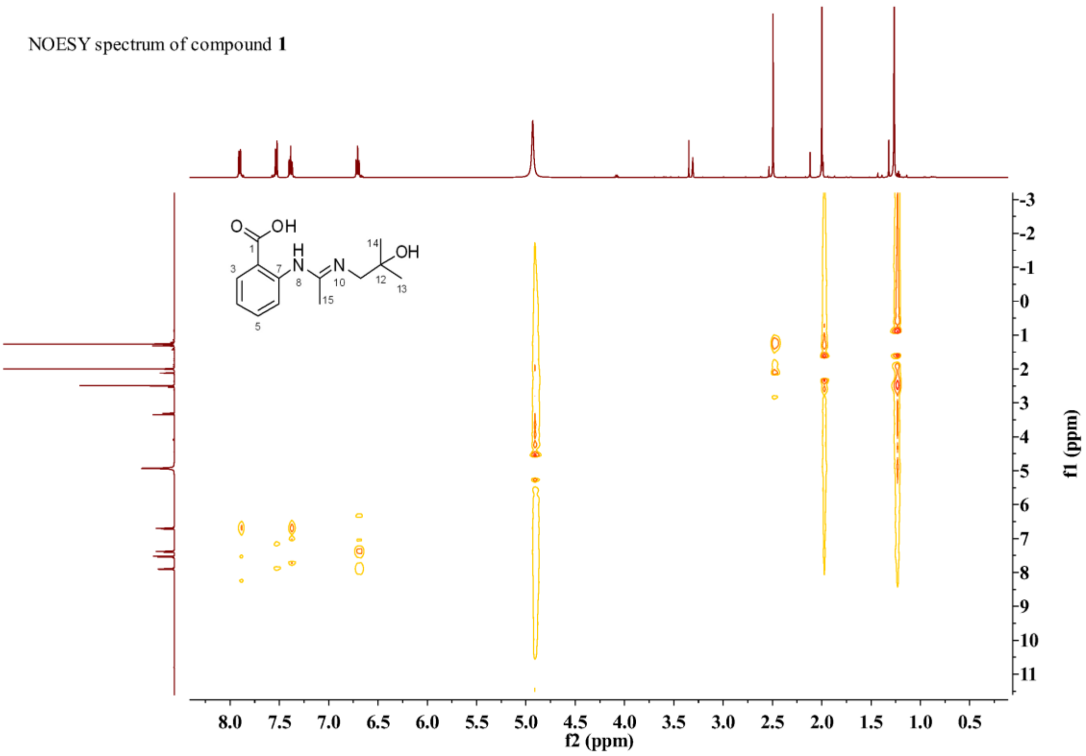

Figure S7. ^{1}H NMR (500 MHz, methanol-d_4) spectrum of compound **2**.

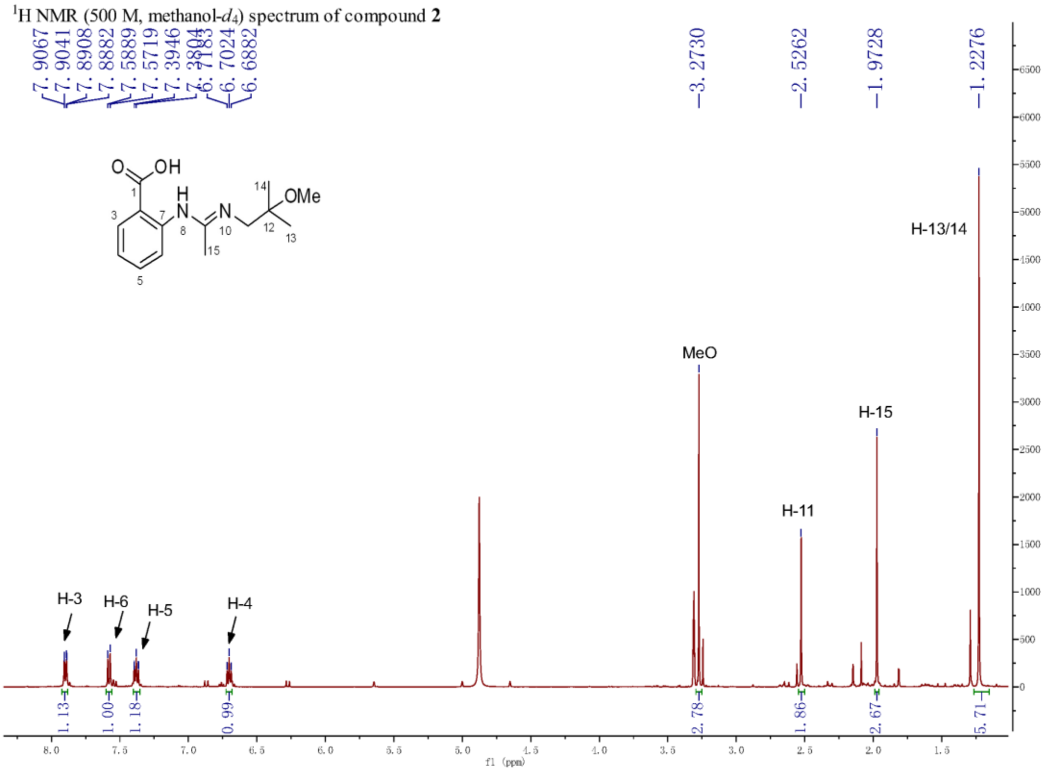

Figure S8. ^{13}C NMR (125 MHz, methanol-d_4) spectrum of compound **2**.

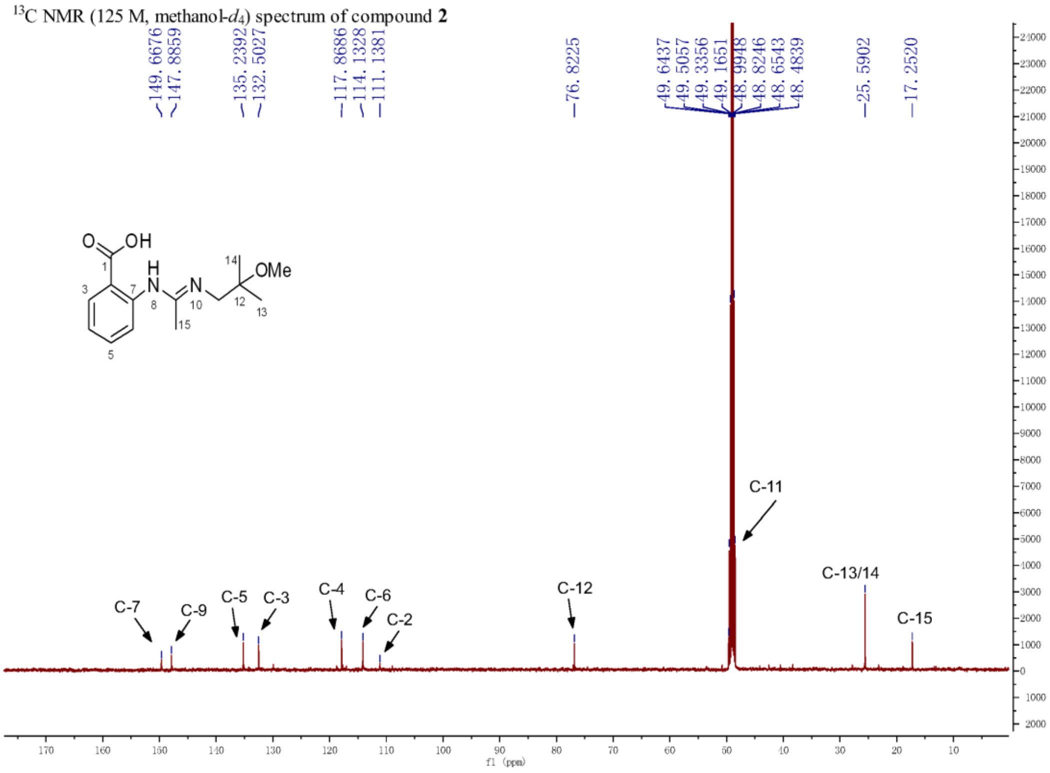

Figure S9. ^{1}H–^{1}H COSY spectrum of compound **2**.

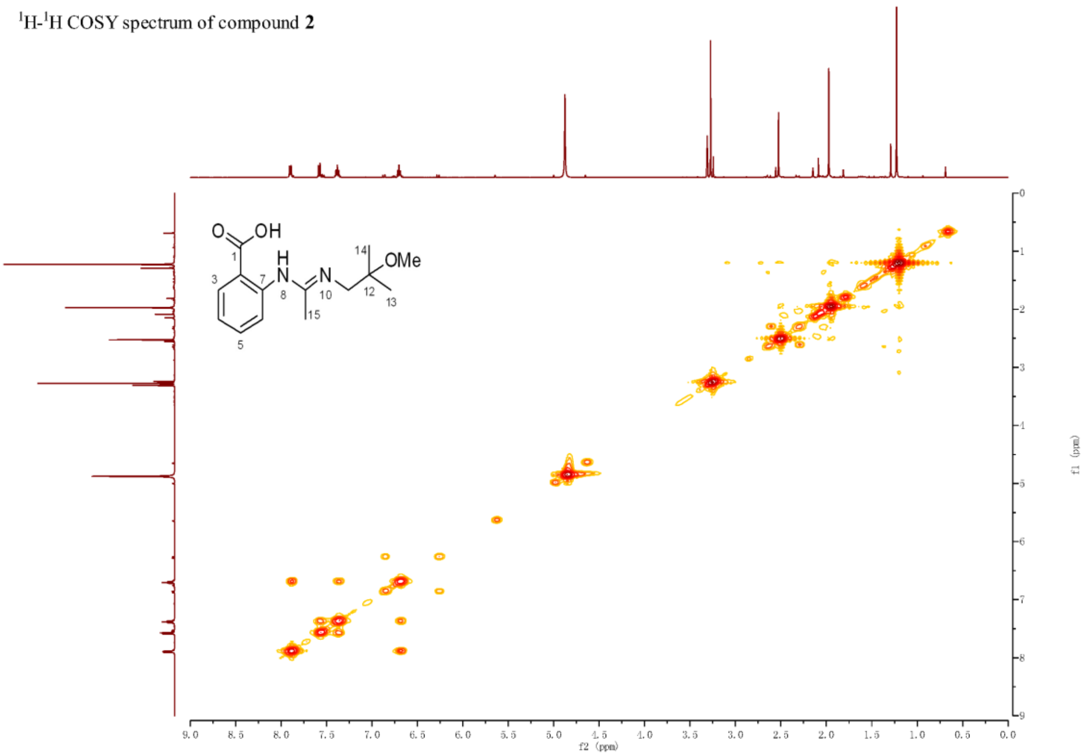

Figure S10. HSQC spectrum of compound **2**.

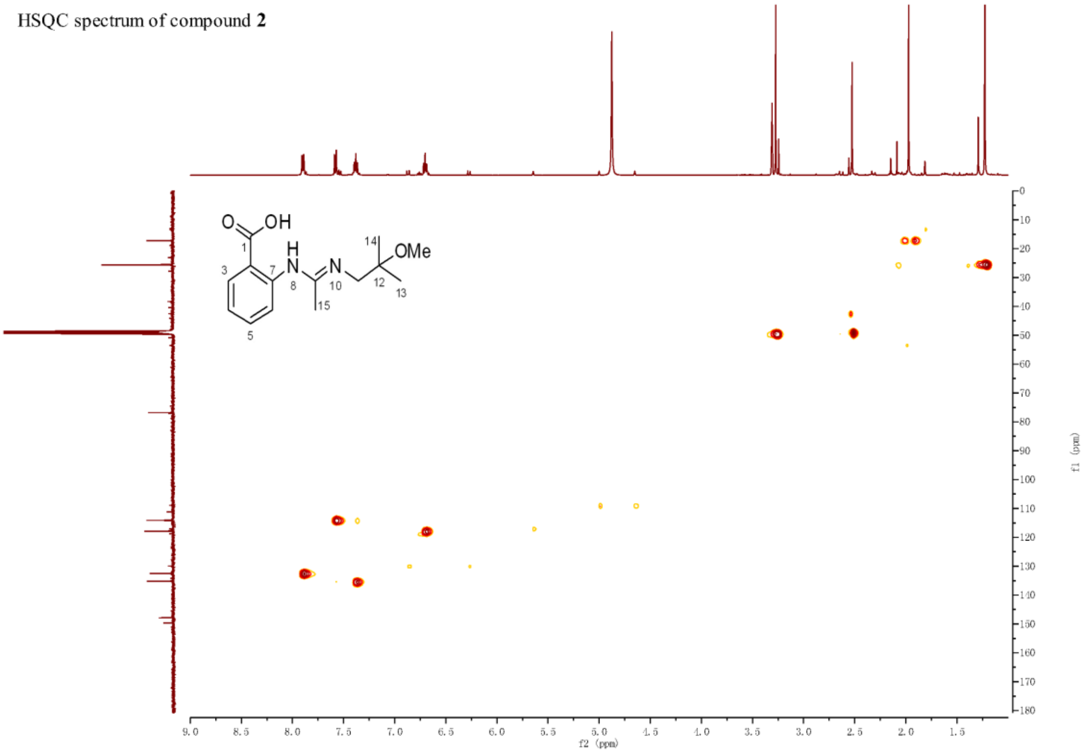

Figure S11. HMBC spectrum of compound **2**.

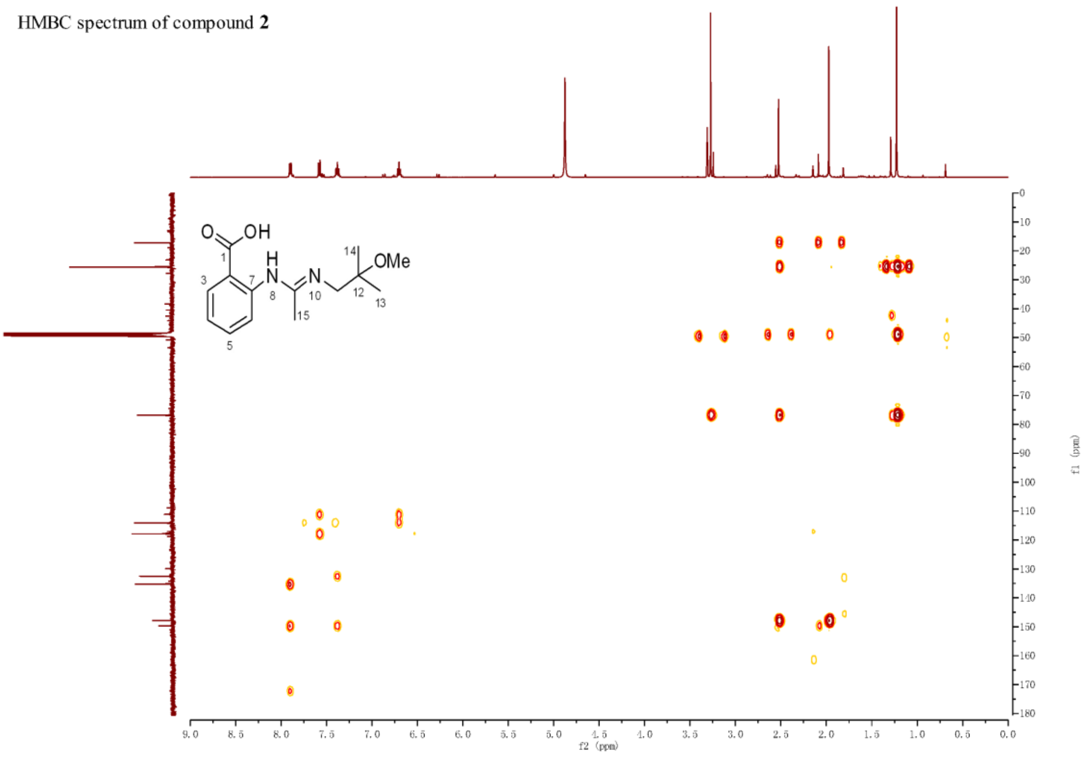

Figure S12. NOESY spectrum of compound **2**.

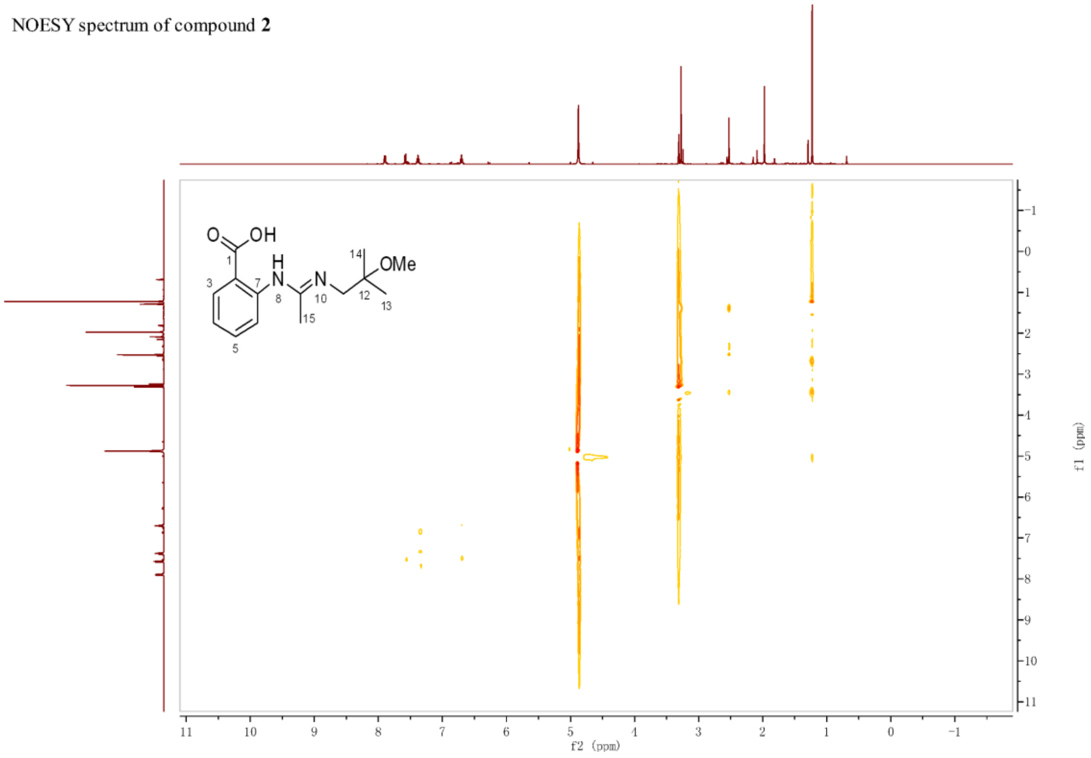

Figure S13. ^{1}H NMR (500 MHz, DMSO-d_6) spectrum of compound **3**.

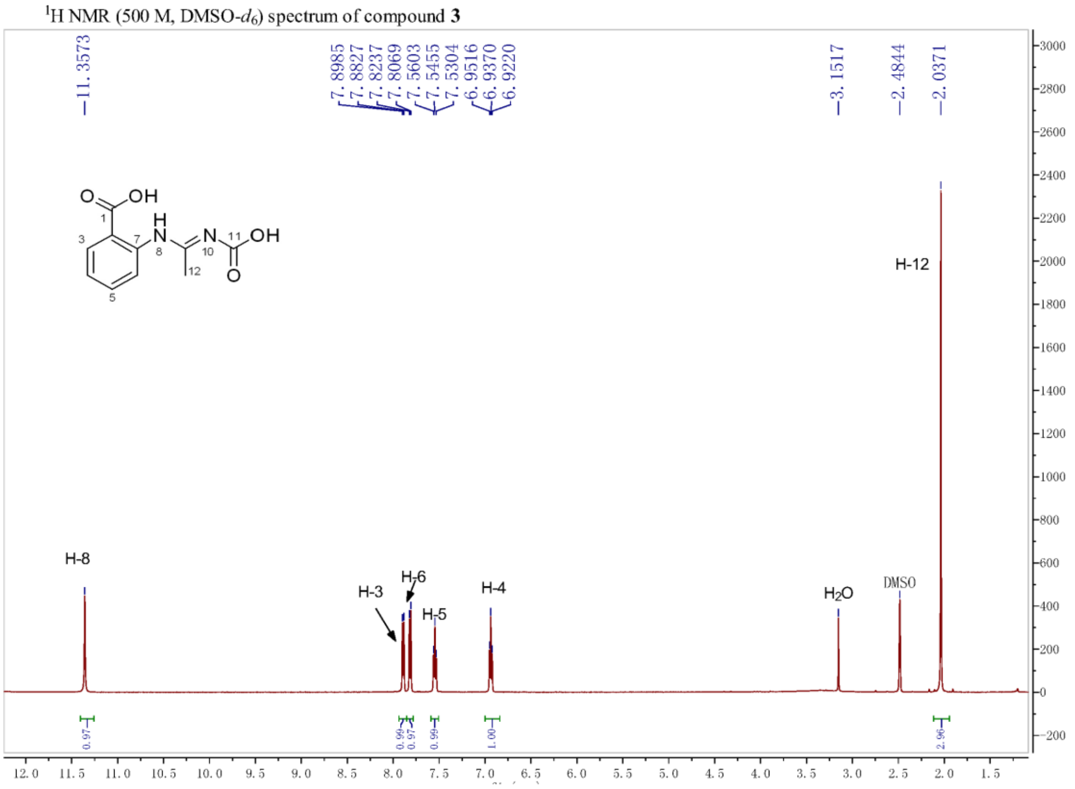

Figure S14. ^{13}C NMR (125 MHz, DMSO-d_6) spectrum of compound **3**.

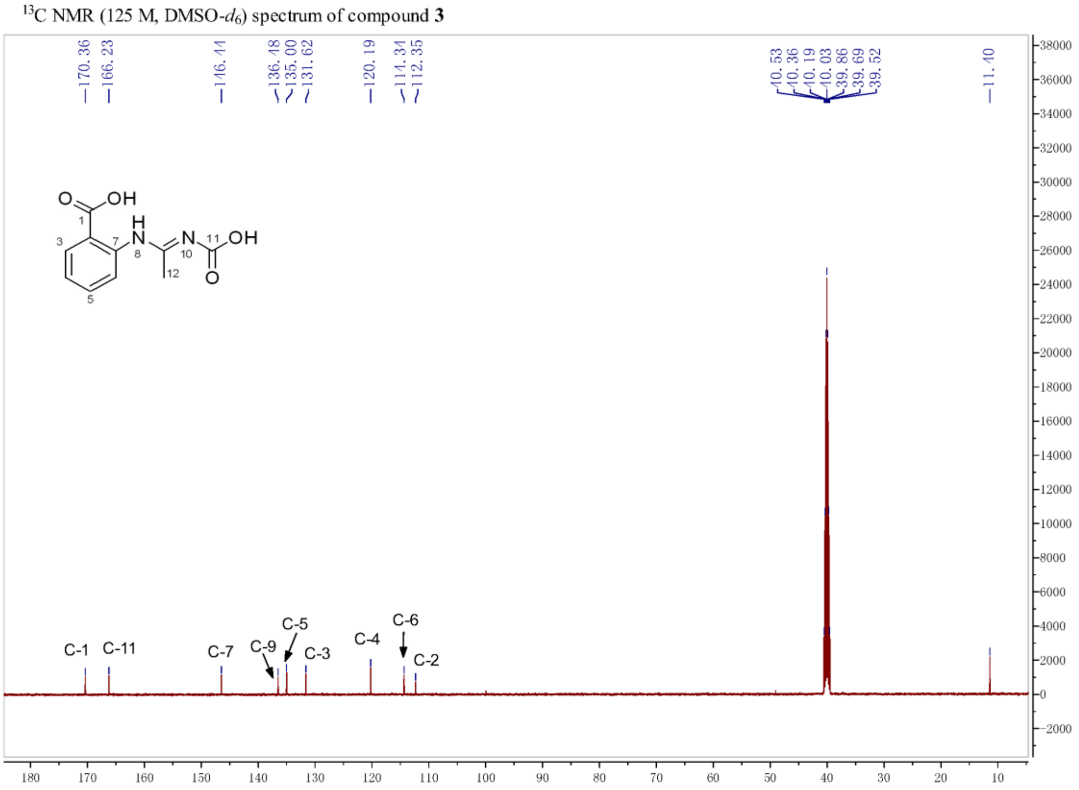

Figure S15. ^{1}H–^{1}H COSY spectrum of compound **3**.

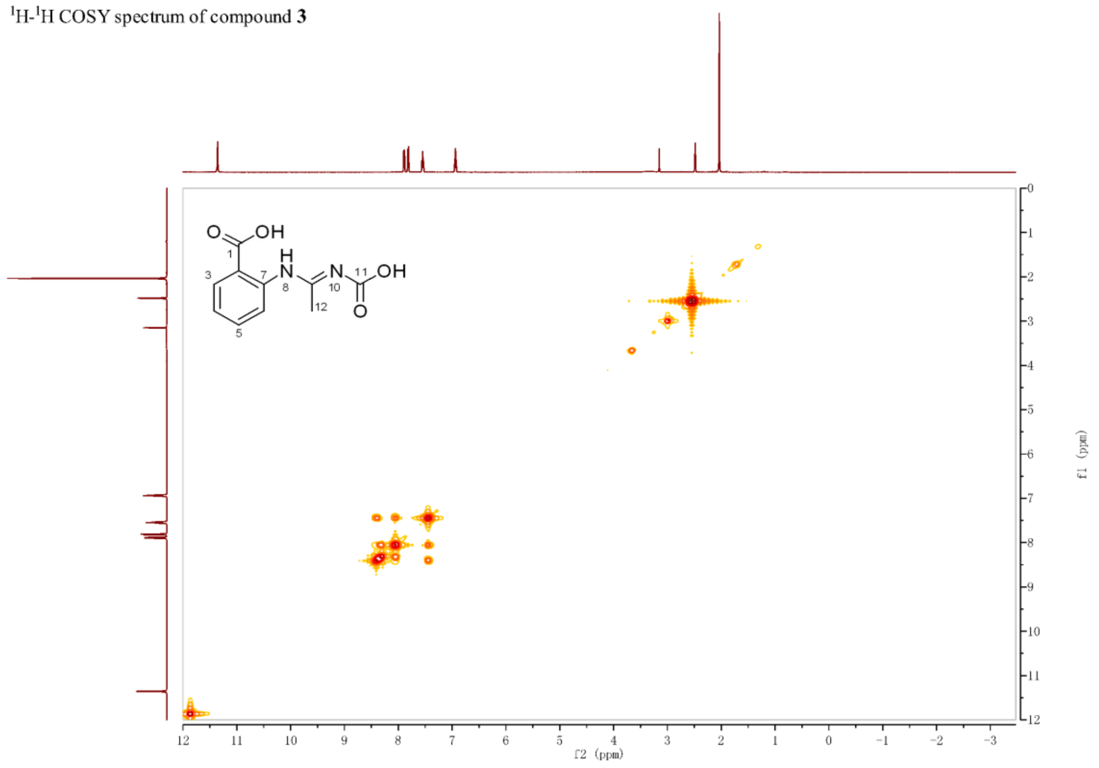

Figure S16. HSQC spectrum of compound **3**.

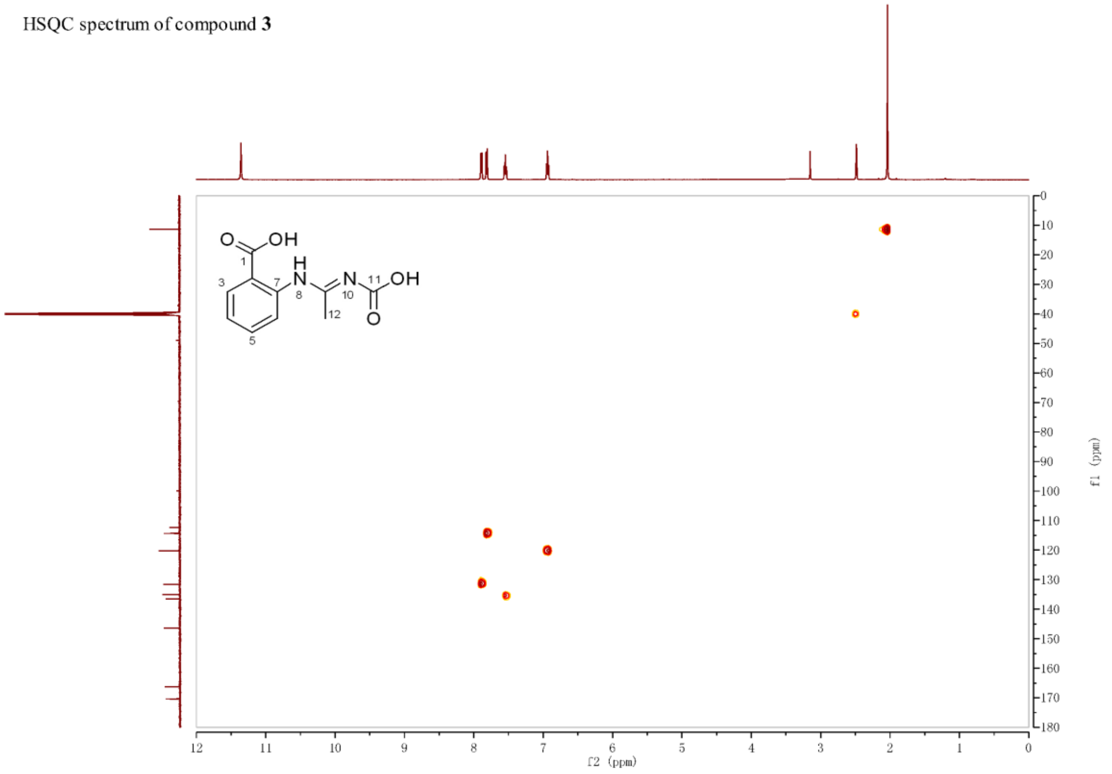

Figure S17. HMBC spectrum of compound **3**.

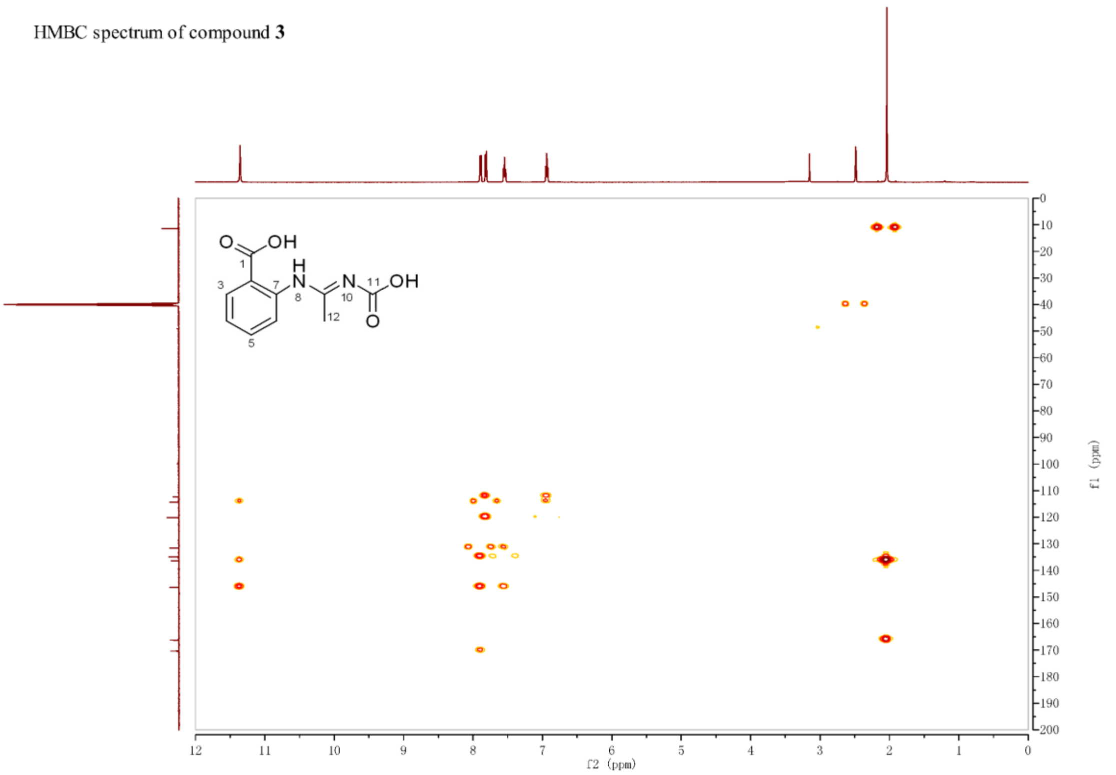

Figure S18. ^{1}H NMR (500 MHz, methanol-d_4) spectrum of compound **4**.

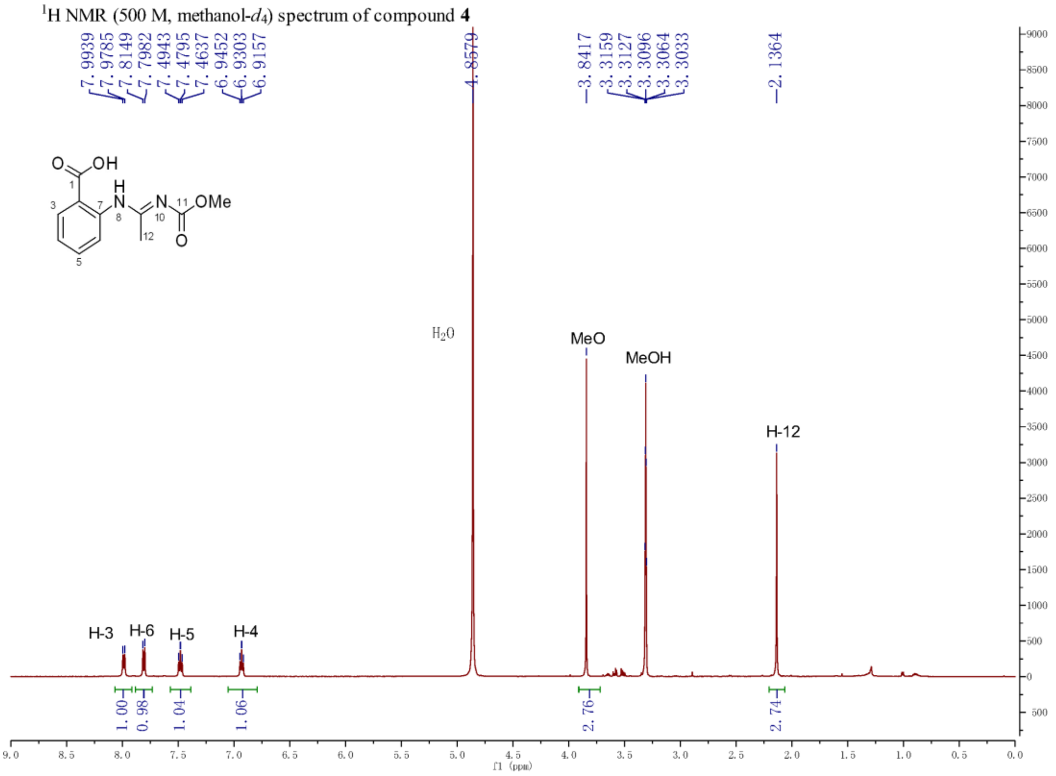

Figure S19. ^{13}C NMR (125 MHz, methanol-d_4) spectrum of compound **4**.

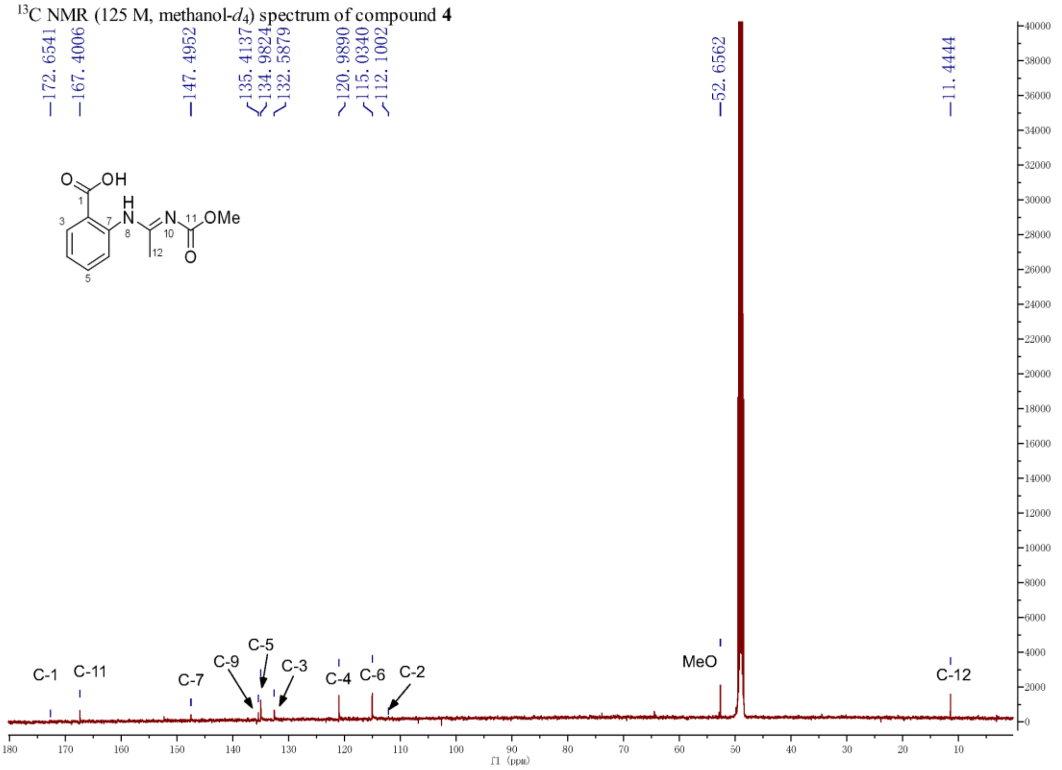

Figure S20. ^{1}H–^{1}H COSY spectrum of compound **4**.

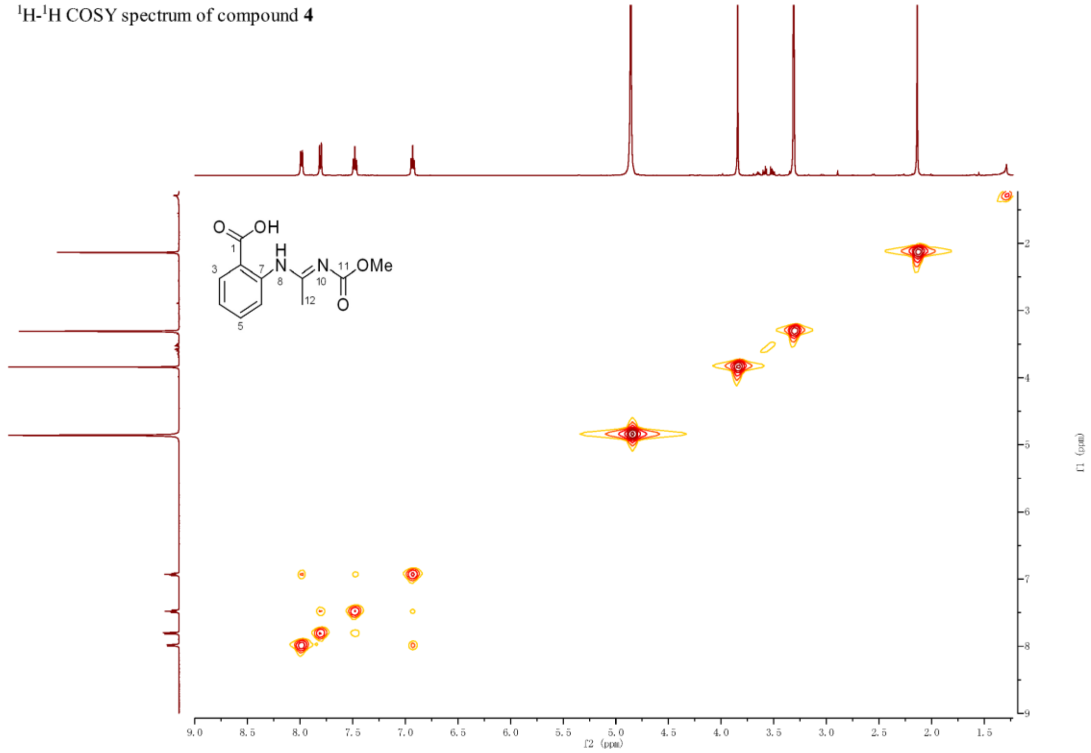

Figure S21. HSQC spectrum of compound **4**.

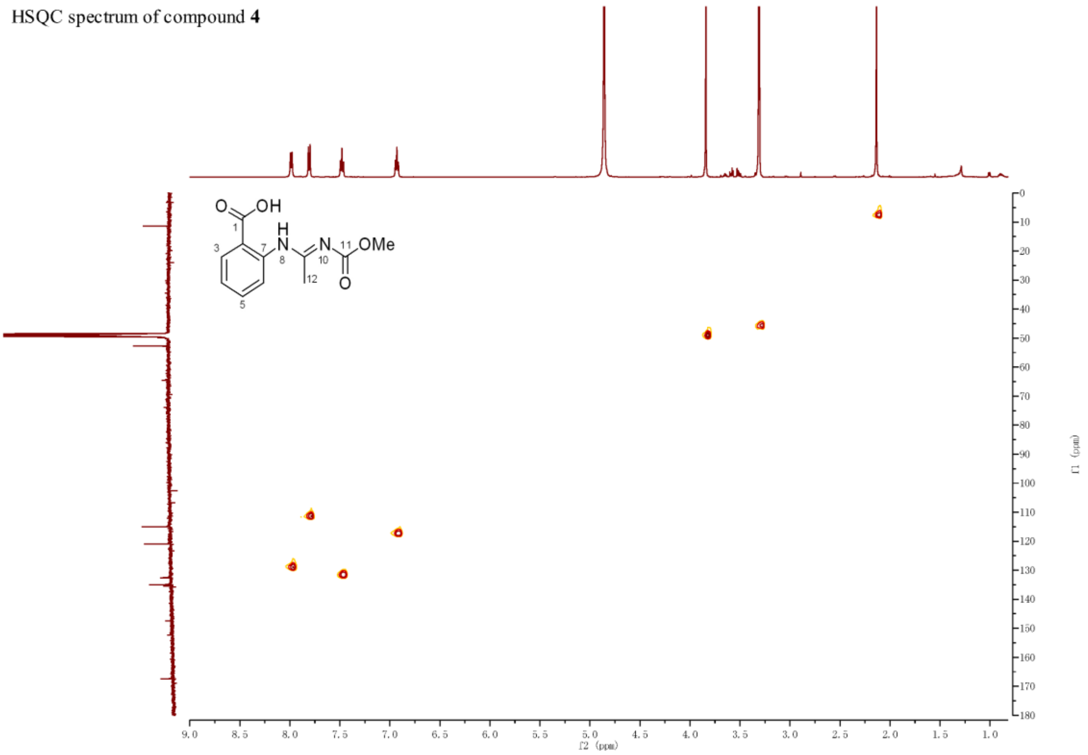

Figure S22. HMBC spectrum of compound **4**.

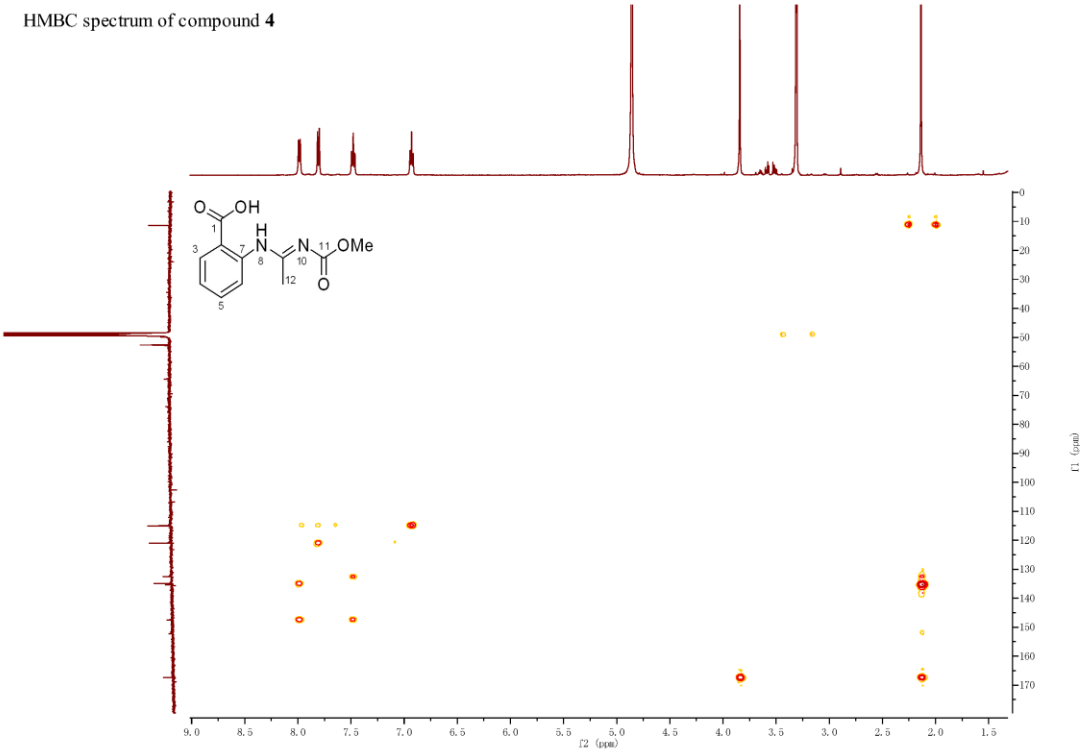

Figure S23. ^{1}H NMR (500 MHz, DMSO-d_6) spectrum of compound **5**.

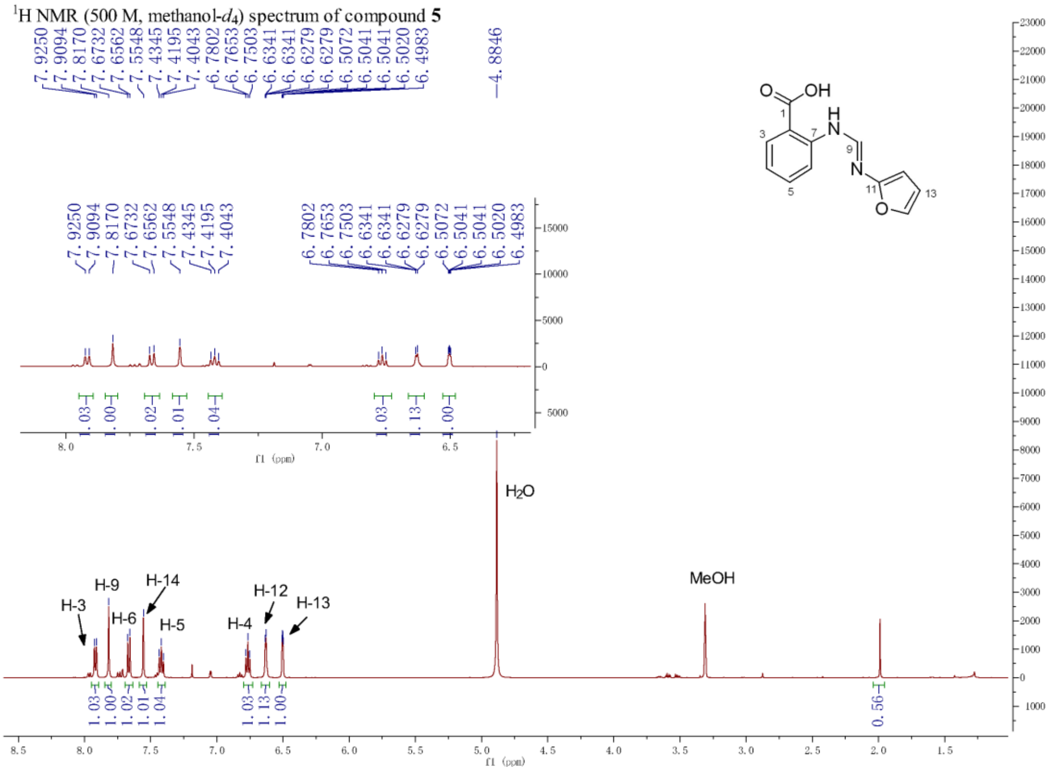

Figure S24. ^{13}C NMR (125 MHz, DMSO-d_6) spectrum of compound **5**.

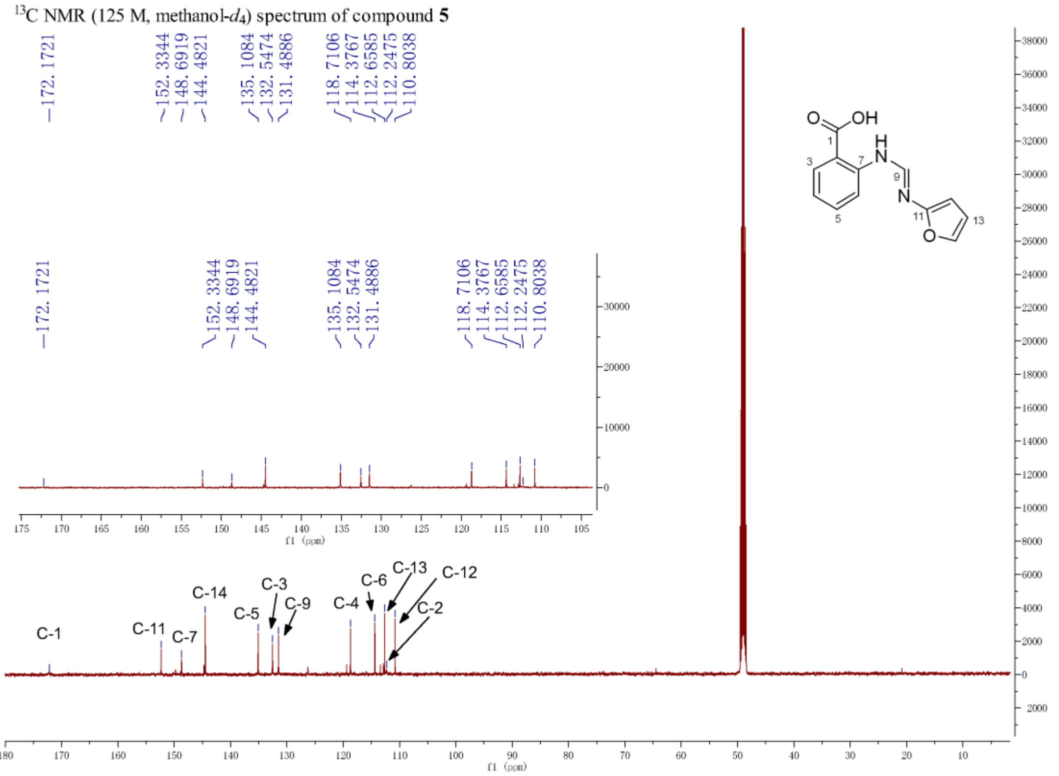

Figure S25. ^{1}H–^{1}H COSY spectrum of compound **5**.

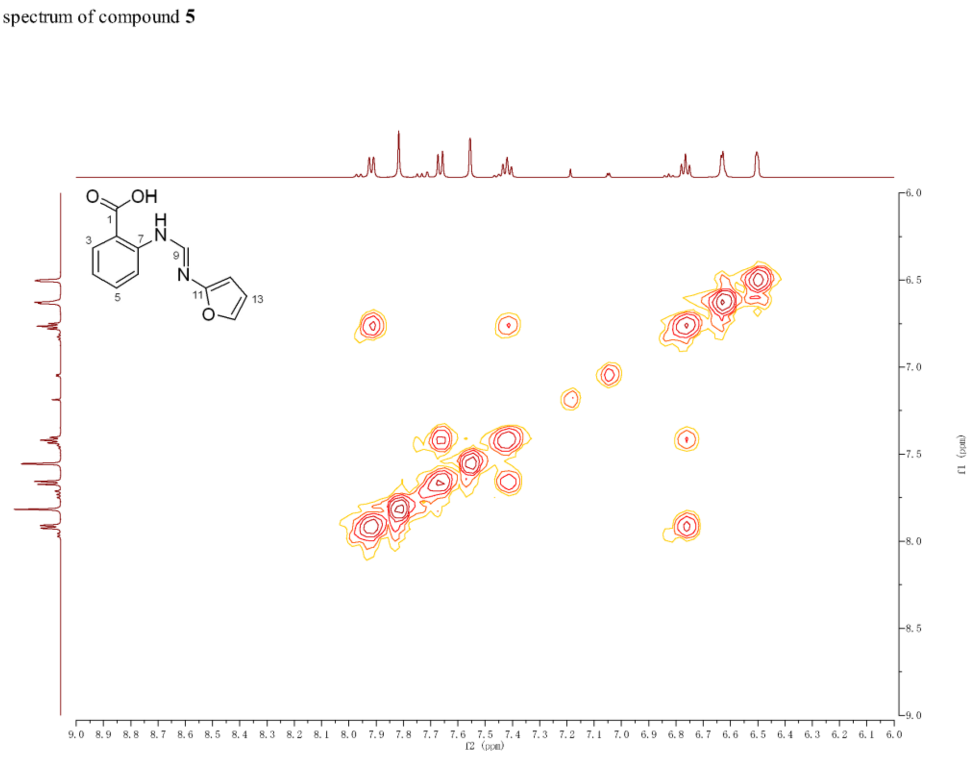

Figure S26. HSQC spectrum of compound **5**.

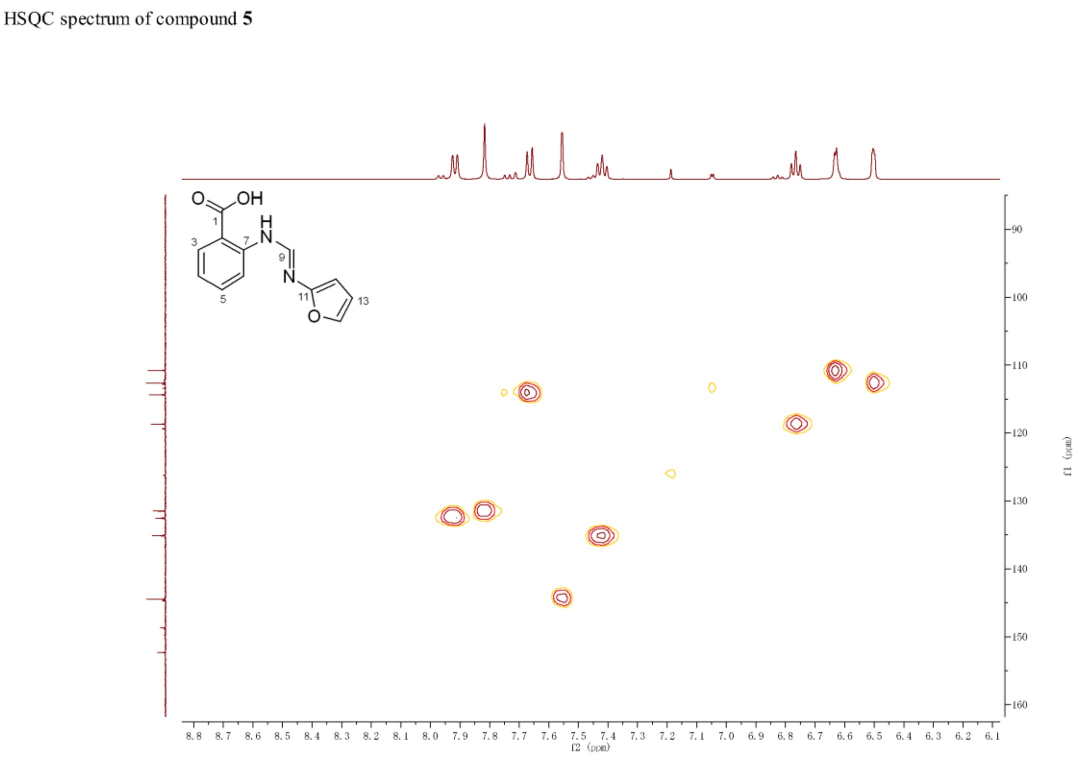

Figure S27. HMBC spectrum of compound **5**.

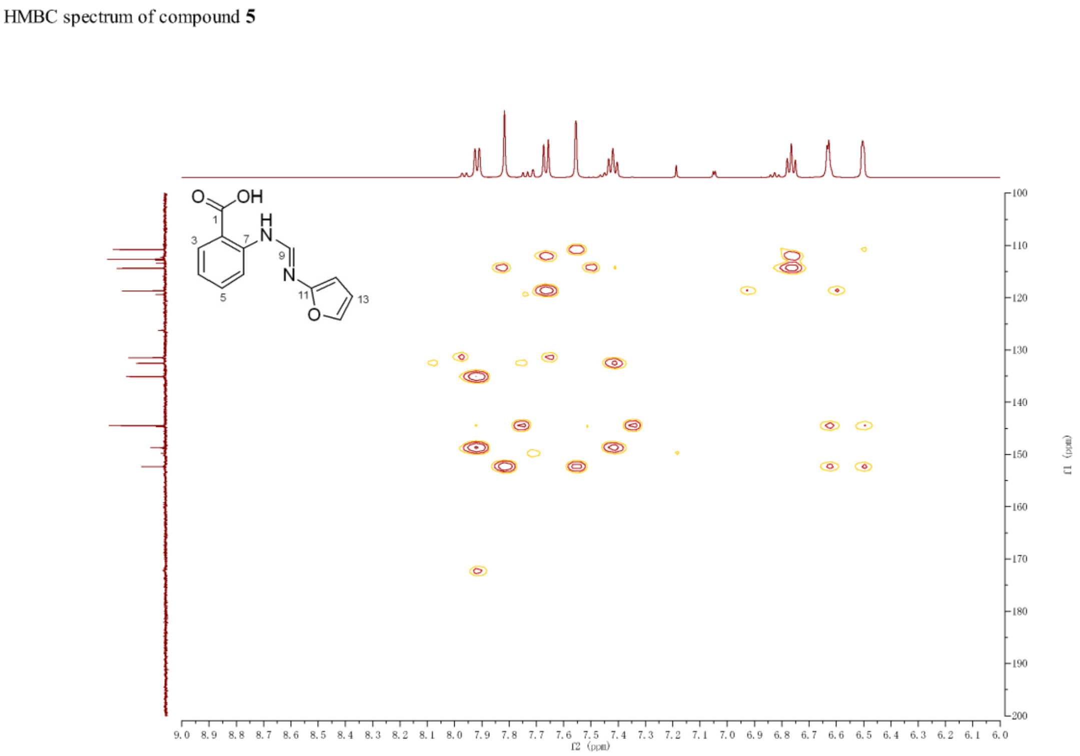

Figure S28. NOESY spectrum of compound **5**.

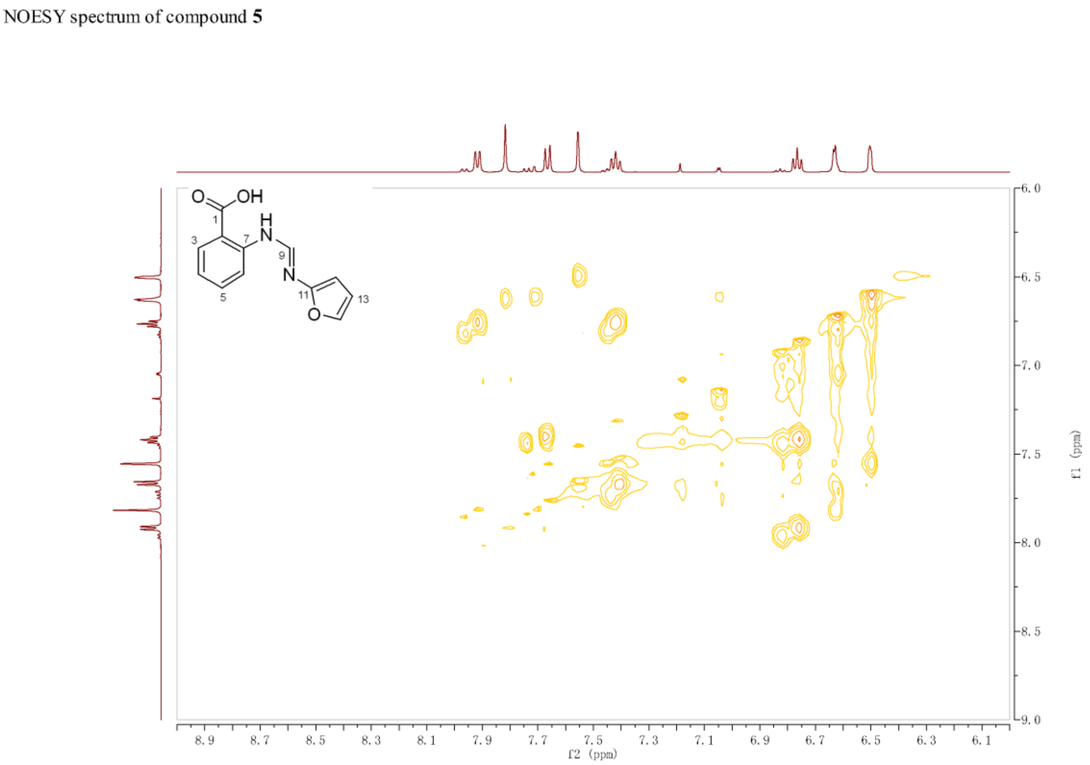

Figure S29. ^{1}H NMR (500 MHz, methanol-d_4) spectrum of compound **6**.

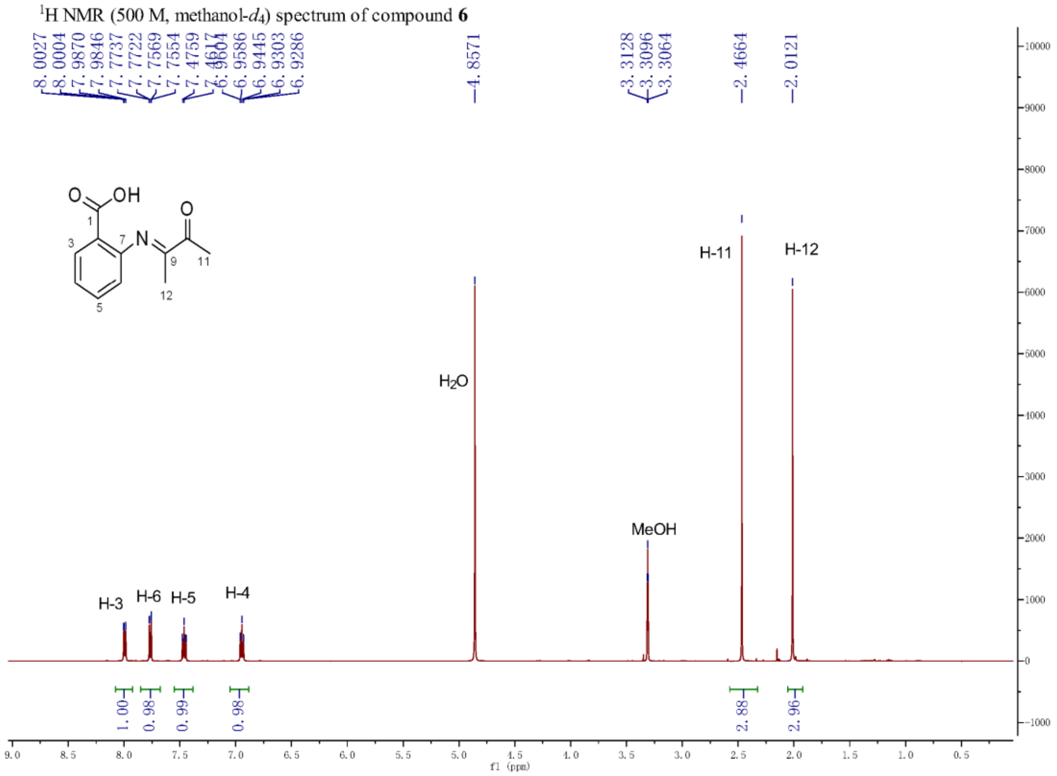

Figure S30. ^{13}C NMR (125 MHz, methanol-d_4) spectrum of compound **6**.

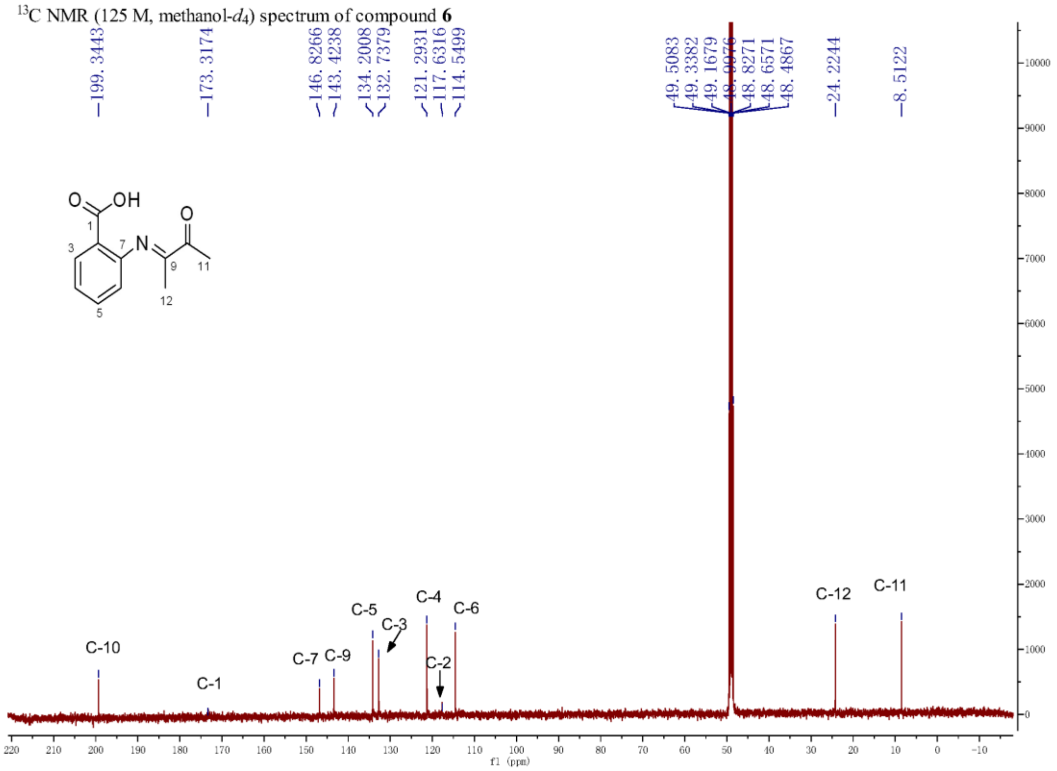

Figure S31. ^{1}H–^{1}H COSY spectrum of compound **6**.

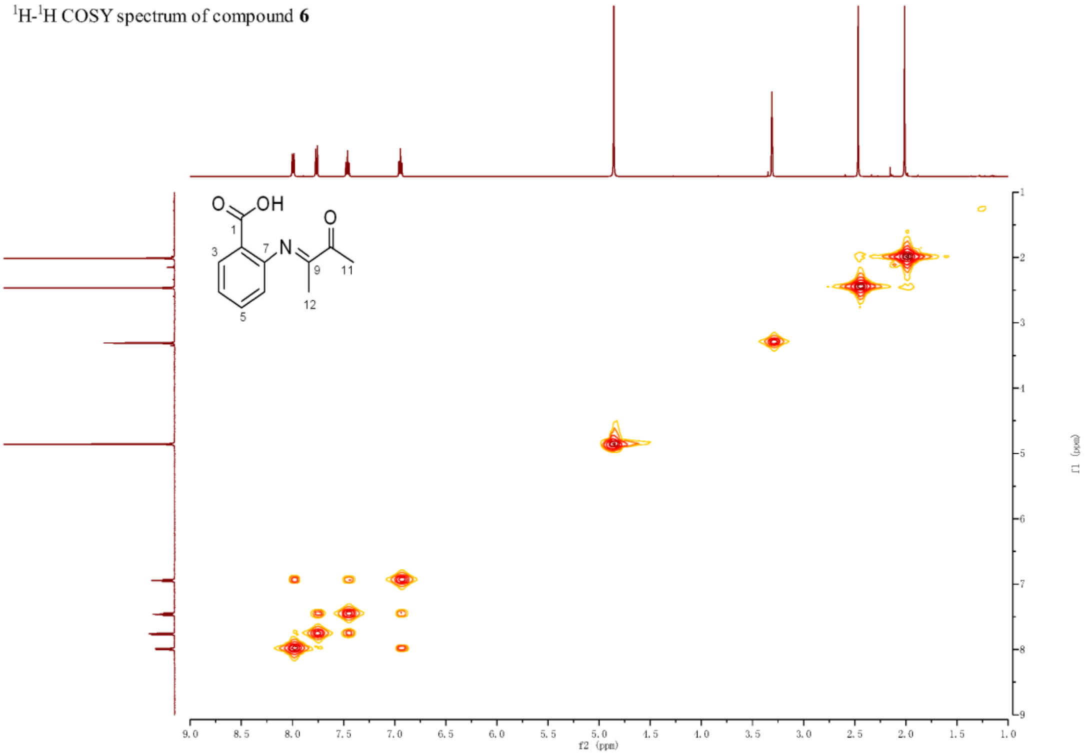

Figure S32. HSQC spectrum of compound **6**.

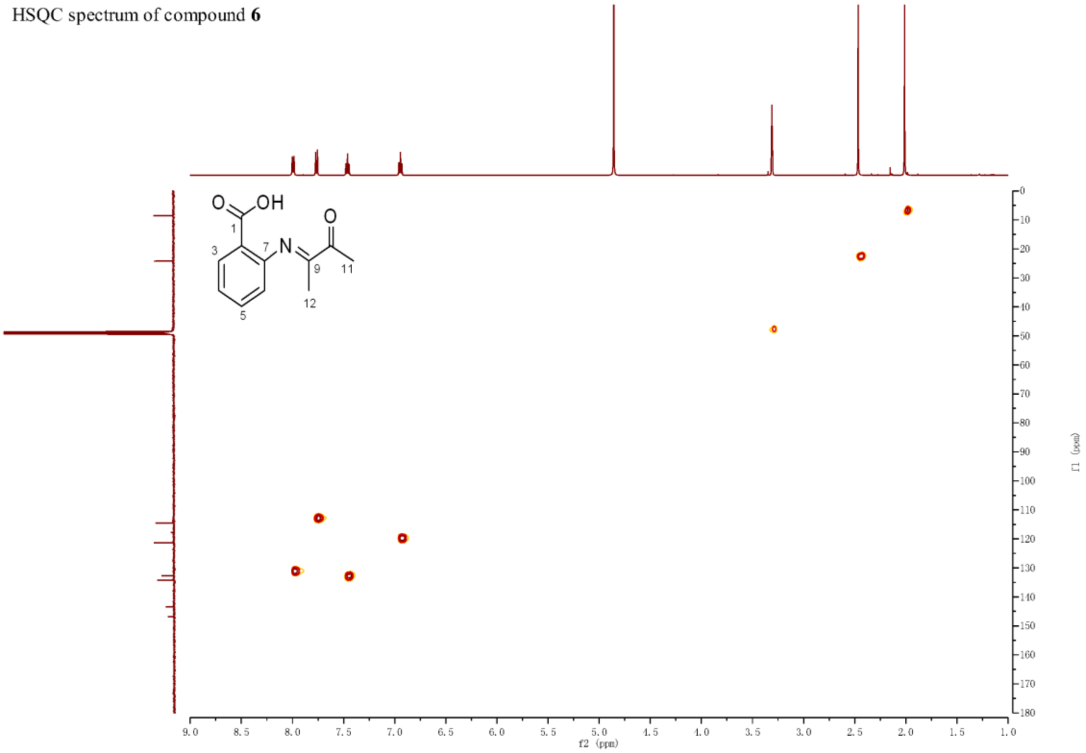

Figure S33. HMBC spectrum of compound **6**.

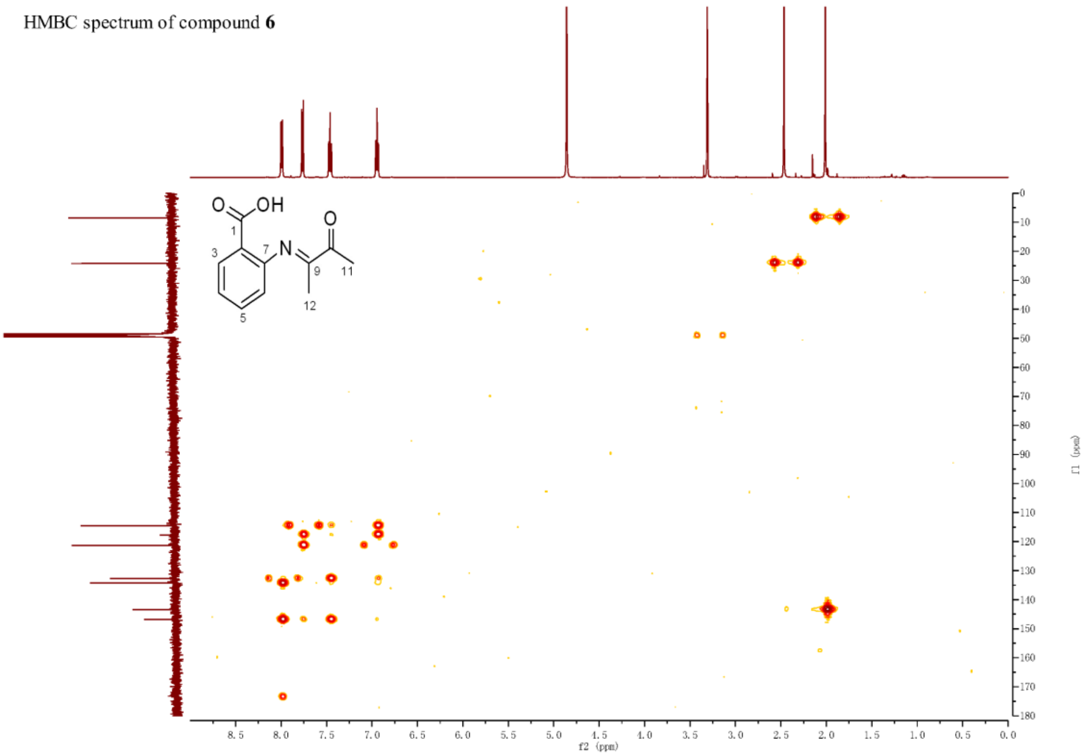

Supplement: Supplementary File 1 — Supplementary Materials (PDF, 1906 KB) [file marinedrugs-11-03068-s001.pdf]
